# A Human–Machine Interaction–Experience–Learning Algorithm for Integrated Lateral–Longitudinal Decision-Making and Control in Intelligent Connected Vehicles

**DOI:** 10.3390/s26196295

**Published:** 2026-10-04

**Authors:** Wenxuan Shen, Cunyue Yan, Zhaoqiang Wang, Zeng Huang, Wei Gao

**Affiliations:** 1 School of Mechanical and Automotive Engineering, Shanghai University of Engineering Science, Shanghai 201620, China; a1076963058@163.com; 2 Shanghai Xizhong Suo Heavy Machinery Complete Set Co., Ltd., Shanghai 201900, China; 19802519573@163.com; 3 The 704th Research Institute of China State Shipbuilding Co., Ltd., Shanghai 200030, China; 13601631225@139.com; 4 Shanghai Hunter Hydraulic Control Technology Co., Ltd., Shanghai 201612, China; 17853311062@163.com

**Keywords:** deep reinforcement learning, Actor–Critic, DDPG-HP, V2X, kalman filtering

## Abstract

**Highlights:**

**What are the main findings?**

- A human-preference reward shaping mechanism is proposed to constrain the policy search space in DRL-based autonomous driving, achieving 1.7 s earlier braking than the DDPG-RRT* baseline.
- A Kalman filter-based V2X communication anomaly compensation module is designed to ensure stable state estimation under packet loss, thereby enhancing the robustness of the system under non-ideal communication conditions.

**What are the implications of the main findings?**

- The integrated lateral–longitudinal decision-making framework enhances pedestrian safety and ride comfort in complex urban traffic scenarios, with collision rate reduced to 0% in dynamic test environments.
- Compared with standard DDPG and DDPG-RRT*, the proposed DDPG-HP algorithm demonstrates faster convergence and better scenario adaptability in the tested scenarios, providing an effective solution for human-like autonomous driving under packet loss and delay conditions.

**Abstract:**

Traditional reinforcement learning for autonomous driving faces challenges such as slow convergence, control instability, and limited adaptability. To address these issues, this paper proposes a hybrid decision-making framework that integrates V2X communication, human–machine interaction experience, and deep reinforcement learning. Based on the Actor–Critic architecture, the framework incorporates a human-like driving tendency network to constrain the policy search space and integrates a Kalman filter-based V2X communication anomaly handling module. The model constructs the vehicle’s action space and state representation. Comparative experiments with a method lacking the driving tendency module and the hierarchical planning method DDPG-RRT* show that DDPG-HP achieves faster convergence and better scenario adaptability than these baselines. It effectively avoids static and dynamic obstacles, demonstrates anticipatory and gradual deceleration, and initiates braking 1.7 s earlier than the DDPG-RRT* method, thereby reducing collision risks and enhancing pedestrian safety. Furthermore, the communication anomaly compensation mechanism effectively improves the system’s adaptability and overall robustness under unstable communication conditions.

## 1. Introduction

With the rapid advancement of intelligent connected and autonomous driving technologies, vehicle decision-making systems are progressively evolving from rule-based static control to data-driven intelligent decision-making. Traditional autonomous driving decision methods are primarily categorized into rule-based and optimization-based approaches. Choi et al. [1] developed decision algorithms suitable for mass-produced vehicles targeting constant-speed cruising and car-following scenarios, yet their applicability is limited in more complex scenarios. Wang et al. [2] proposed a cooperative adaptive cruise control strategy based on distributed model predictive control. Their approach reduces fluctuations in platoon following distance and significantly improves stability through hierarchical controller design and robust optimization. Although these methods offer structural clarity and reliability, they often lack flexibility and generalization in complex and dynamic environments.

Deep reinforcement learning has shown significant potential in autonomous driving. It can learn and optimize decisions autonomously in complex environments. However, pure reinforcement learning algorithms face challenges such as extended training periods, convergence difficulties, and limited interpretability of policy behavior. Wu et al. [3] introduced a multi-vehicle cooperative driving strategy based on V2X communication, integrating vehicular network information through reinforcement learning to enhance environmental perception and decision safety. Qiu et al. [4] employed maximum entropy inverse reinforcement learning to construct a driving style-aware car-following model, effectively balancing efficiency and safety. Nevertheless, existing research still exhibits limitations in areas such as human driving behavior modeling and multi-objective collaborative optimization. For instance, mainstream algorithms like DDPG encounter challenges in training stability and sample efficiency. Although Liu et al. [5] proposed the CLPER-DDPG algorithm, which improves longitudinal control stability through curriculum learning and prioritized experience replay mechanisms, its adaptability to lateral coordination and dynamic interactive scenarios remains insufficient.

To address decision robustness and safety issues in complex dynamic interactive environments, researchers have begun exploring hybrid architectures and collaborative optimization methods. Wei et al. [6] investigated the impact of different driving styles on rear-end collision risk. Their analysis revealed differences in reaction patterns between aggressive and conservative drivers in emergency braking scenarios, providing important references for the design of active safety systems. Islam et al. [7] utilized driver data to construct reward functions, achieving driver-like longitudinal decision-making. Liu et al. [8] proposed a deep reinforcement learning-based autonomous drift system. By combining a cubic spline curve-based drift path planner and a Refined-exploration Soft Actor Critic algorithm, it achieves robust obstacle avoidance capabilities under extreme driving conditions. While these methods have improved decision performance at various levels, they still lack systematic integration in areas such as the fusion of human–machine interaction experience and lateral–longitudinal collaborative optimization. Li et al. [9] proposed a human-like driving decision method for unsignalized intersections based on a game-theoretic framework. By quantifying driver payoff through Prospect Theory and introducing a probabilistic model to describe driver acceleration tendencies, it ensures a high success rate of safe interactions while improving traffic efficiency. Ma et al. [10] proposed a DDPG algorithm that accelerates policy convergence through expert experience pre-training.

However, existing approaches often focus on single tasks or isolated modules, lacking systematic integration of driving experience, environmental interaction, and multi-objective collaboration. This paper proposes a lateral–longitudinal collaborative decision-making and control framework that integrates human–machine interaction experience with deep reinforcement learning. By constructing a driving preference-guided reward mechanism, a hierarchical experience storage and learning module, and a communication anomaly robustness compensation mechanism, the framework aims to achieve safe, efficient, and human-like autonomous driving decisions in dynamic traffic scenarios. Performance is evaluated through comparisons with a method lacking the driving tendency module and the hierarchical planning-based DDPG-RRT*.

The main contributions of this paper include the following: (1) designing a network that incorporates human driving preferences to enhance the human-likeness and comfort of the policy; (2) constructing a lateral–longitudinal collaborative action space and state representation to achieve multi-objective collaborative optimization, including pedestrian courtesy and proactive braking obstacle avoidance; (3) introducing a communication packet loss compensation module to enhance the system’s robustness under non-ideal V2X environments.

## 2. Deep Reinforcement Learning Algorithm

### 2.1. Vehicle Model

**Figure 1:**
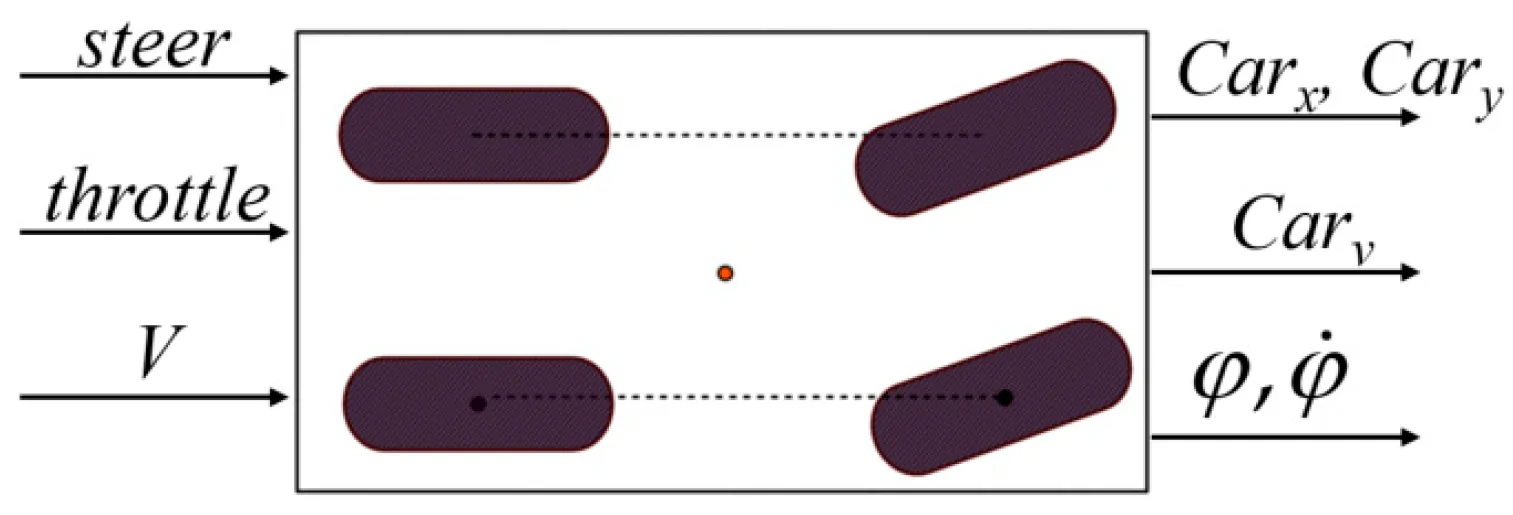
Two-degree-of-freedom Bicycle Model.

This system primarily consists of four major components: the agent module [11], environmental interaction, state estimation with experience storage and learning mechanism, and a communication packet loss compensation module [12]. This paper first establishes a two-degree-of-freedom vehicle dynamics model using steering wheel angle, throttle/brake inputs, and initial vehicle speed as control inputs. The model outputs the vehicle’s position, velocity, and yaw rate, along with sensor-detected obstacle positions, as well as pedestrian positions and velocities. This approach reduces the state dimension while effectively capturing the vehicle’s steering dynamics, as illustrated in Figure 1.

This paper proposes an end-to-end autonomous driving decision-making and control framework based on an improved DDPG [13] algorithm, aiming to address safe driving challenges in complex and dynamic traffic scenarios. The method builds upon the 2-DOF vehicle dynamics model and takes the target steering wheel angle, throttle command, brake command, and initial vehicle state as inputs, and it outputs the vehicle speed and yaw rate. These outputs are combined with high-definition map data to compute the vehicle’s lateral and longitudinal position information.

### 2.2. DDPG-HP Algorithm Framework

To enhance the robustness of the system in real-world environments, a communication anomaly module is integrated into the framework. This module employs a Kalman filter [14] for signal filtering and compensation, addressing disruptions such as V2X communication [15] link interruptions or delays. The aim is to generate control strategies that balance efficiency and safety, enabling the system to output reasonable control actions in real time in complex traffic scenarios.

**Figure 2:**
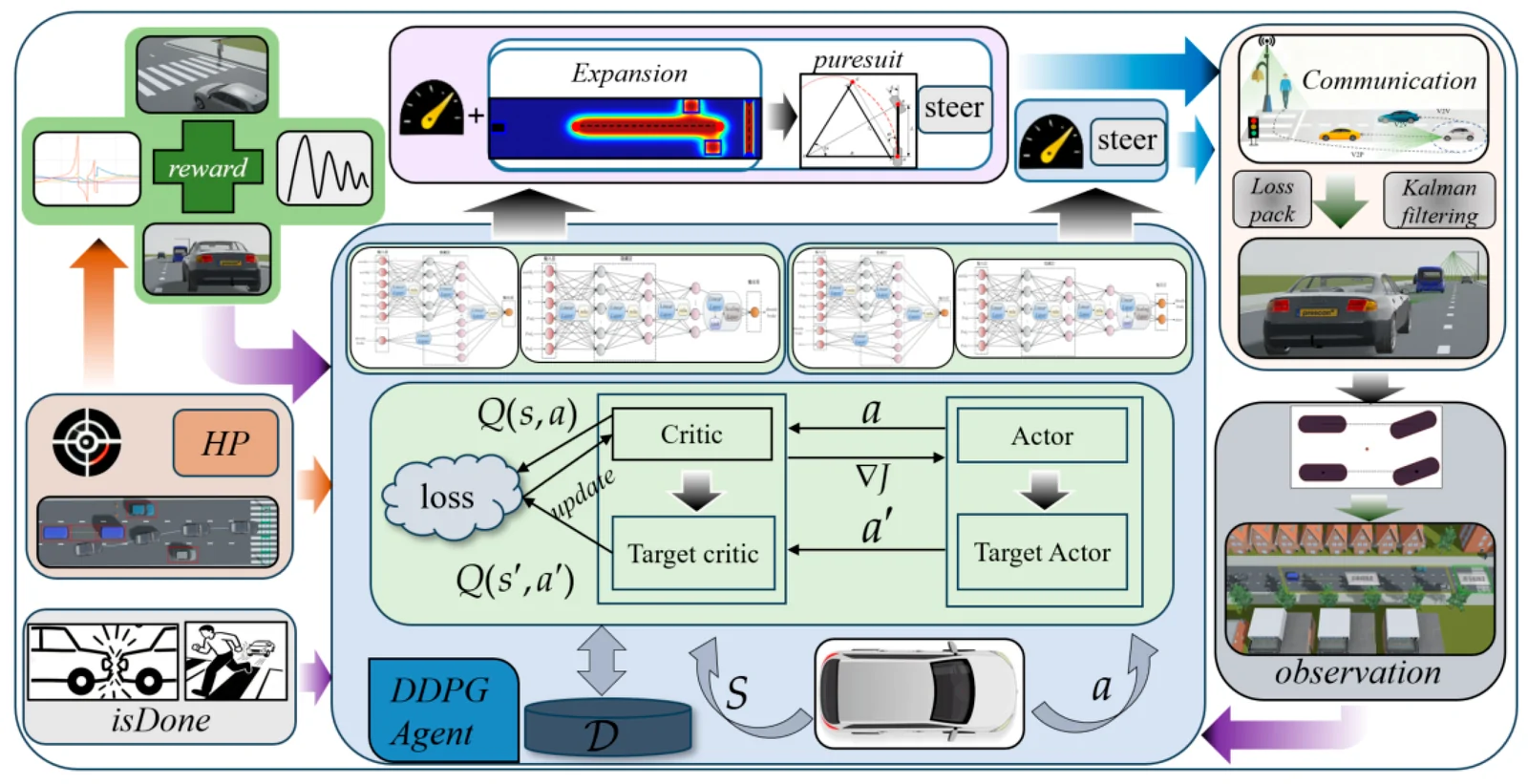
Overall architecture of the safe and efficient self-evolving algorithm integrating human driving tendency and V2X.

This framework introduces the concept of “Human Preference.” Through continuous online learning and parameter iteration, it generates guiding signals that better reflect natural human driving habits and rational decision-making. These signals are integrated into the reinforcement learning optimization process, allowing the algorithm to converge rapidly within a relatively short training period toward control strategies that better reflect human driving intuition while balancing safety and traffic efficiency. The overall algorithm follows the dual-network Actor–Critic architecture, modeling the decision-making and control problem in autonomous driving as a holistic optimization task within a continuous action space. This creates an end-to-end autonomous driving system integrating perception, decision-making, control, and learning. To validate the effectiveness and advantages of the proposed method, this study also introduces the classic path planning algorithm RRT* as a performance benchmark. The framework is illustrated in Figure 2.

## 3. Agent Module

This study constructs an autonomous driving system framework based on the DDPG-HP algorithm, integrating perception, decision-making, control, and learning modules. The overall system architecture comprises four main components: the agent module, the environment interaction module, the state estimation and experience replay mechanism, and the communication packet loss compensation module.

A DDPG agent based on the Markov Decision Process (MDP) [16] is developed to enable vehicle trajectory tracking and collaborative decision-making in complex dynamic traffic environments. The state space *s* is defined as multi-source perceptual information, including the lateral and longitudinal positions and velocities of the ego vehicle, obstacle vehicles, and pedestrians. The action space consists of continuous adjustable control inputs such as throttle, brake, and steering wheel angle. The reward function is designed to balance multiple objectives, including safety, efficiency, and comfort. Through iterative training across diverse environmental scenarios, the DDPG agent can approximate the optimal control policy, demonstrating fast convergence, smooth control outputs, and strong generalization capabilities in continuous action spaces. This makes it particularly suitable for predictive deceleration and steering coordination in dynamic interactive scenarios such as pedestrian crossings.

### 3.1. DDPG-HP Pseudocode

This module adopts the Deep Deterministic Policy Gradient (DDPG) algorithm to build an agent for end-to-end control policy learning. Combining the advantages of policy gradient methods and value function approximation, DDPG is well-suited for continuous action space control problems in autonomous driving. The module includes four core neural networks: the Actor network, Critic network, Target–Actor network, and Target–Critic network. The pseudocode of DDPG-HP is presented in Algorithm 1.
**Algorithm 1** Pseudocode of the DDPG-HP Algorithm.**Input:** State observation s, sampling time *T_s_* = 0.02 s, total duration *T_f_* = 20 s **Random initialization:** Critic network *Q*(*s*, *a*|*θ^c^*) and Target-Critic network *Q*′(*s*, *a*|*θ^c^*′) Actor network *μ*(*s* |*θ^μ^*) and Target-Actor network *μ*′(*s*|*θ^μ^*′) Experience replay pool *D* with capacity = 10^6^, minibatch = 128 OU noise: initial variance = 1.2, variance decay rate = 1 × 10^−6^ **Training loop:** For episode = 1 to *N*_max_ do:    Initialize environment state *s* = [*x*_car_, *y*_car_, *v*_car_, …, *x_p_*, *y_p_*, *v_p_*]    For *t* = 1 to ceil (*T_f_*/*T_s_*) do:        Select action *a_t_* based on current policy and OU noise        Execute action *a_t_* = [t*h*rottle/brake, steeR]*^T^*        Obtain reward *r_t_* and next state *s_t_*_+1_        Store experience (*s_t_*, *a_t_*, *r_t_*, *s_t_*_+1_) into replay pool D        If number of samples in D ≥ 128 then:            Randomly sample 128 experiences as minibatch (*s_i_*, *a_i_*, *r_i_*, *s_i_*_+1_)            Compute TD error *δ_i_*;            Update Critic network parameters: $\theta^{c}\leftarrow \theta^{c}+\alpha_{c}\nabla_{\theta^{c}}L(\theta^{c})$            Update Actor network parameters: $\theta^{\mu }\leftarrow \theta^{\mu }+\alpha_{a}\nabla_{\theta^{\mu }}J(\theta^{\mu })$            Soft update target networks: $$\theta^{c^{\prime }}\leftarrow \tau \theta^{c}+(1-\tau )\theta^{c^{\prime }}\;\;\;\theta^{\mu^{\prime }}\leftarrow \tau \theta^{\mu }+(1-\tau )\theta^{\mu^{\prime }}$$            End        End        If convergence criterion is met then            Stop training        End End **Return:** Trained Actor network *μ*(*s*|*θ^μ^*)

The training process adopts an empirical convergence criterion: training is considered converged when the average reward changes by less than ±2% over 100 consecutive episodes, at which point training is manually terminated. The maximum loop bound *N*_max_ is set to 6500 episodes as a safeguard.

The dual-network Actor–Critic architecture ensures control precision in continuous action spaces and provides a flexible interface for integrating the driving tendency model and reward shaping.

### 3.2. Space Design

To address the task requirements of coordinating lateral and longitudinal control for autonomous vehicles during motion, this paper proposes a dynamic decision-making framework that incorporates a driving tendency network to achieve safe obstacle avoidance in complex environments. Specifically, in pedestrian crossing zones such as zebra crossings, to ensure pedestrian safety, the system state space must simultaneously account for the ego vehicle’s motion state, the dynamics of surrounding vehicles, and pedestrian behavior information. Based on this, the state space is designed as follows:

(1) $$S=[x_{\mathrm{car}},y_{\mathrm{car}},v_{\mathrm{car}},x_{obs1},y_{obs1},v_{obs1},\ldots ,x_{obsn},y_{obsn},v_{obsn},x_{p},y_{p},v_{p}]$$

where *x*_car_, *y*_car_, and *v*_car_ represent the longitudinal coordinate, lateral coordinate, and velocity of the ego vehicle; each obstacle-vehicle is described by a triplet of position-velocity variables *x*_obs_, *y*_obs_, and *v*_obs_; and *x_p_*, *y_p_*, and *v_p_* indicate the position, velocity, and heading angle of the pedestrian. Here, *n* denotes the number of obstacle vehicles relevant to the ego vehicle’s trajectory. In the current network architecture, the input dimension is fixed, and *n* is set to a predefined maximum value *n*_max_. This value is determined by the actual scenario and can be adjusted as needed. When the actual number of obstacle vehicles is fewer than *n*_max_, zero padding is applied; when it exceeds *n*_max_, only the closest vehicles are retained. This fixed-dimensional design with padding/truncation is common in standard MLP-based reinforcement learning, but it may become less flexible in highly dense traffic where the number of surrounding agents changes dynamically. Attention mechanisms or graph neural networks (GNNs) could process a variable number of agents more systematically, which is an important direction for future work.

### 3.3. Neural Network Architecture Design

**Figure 3:**
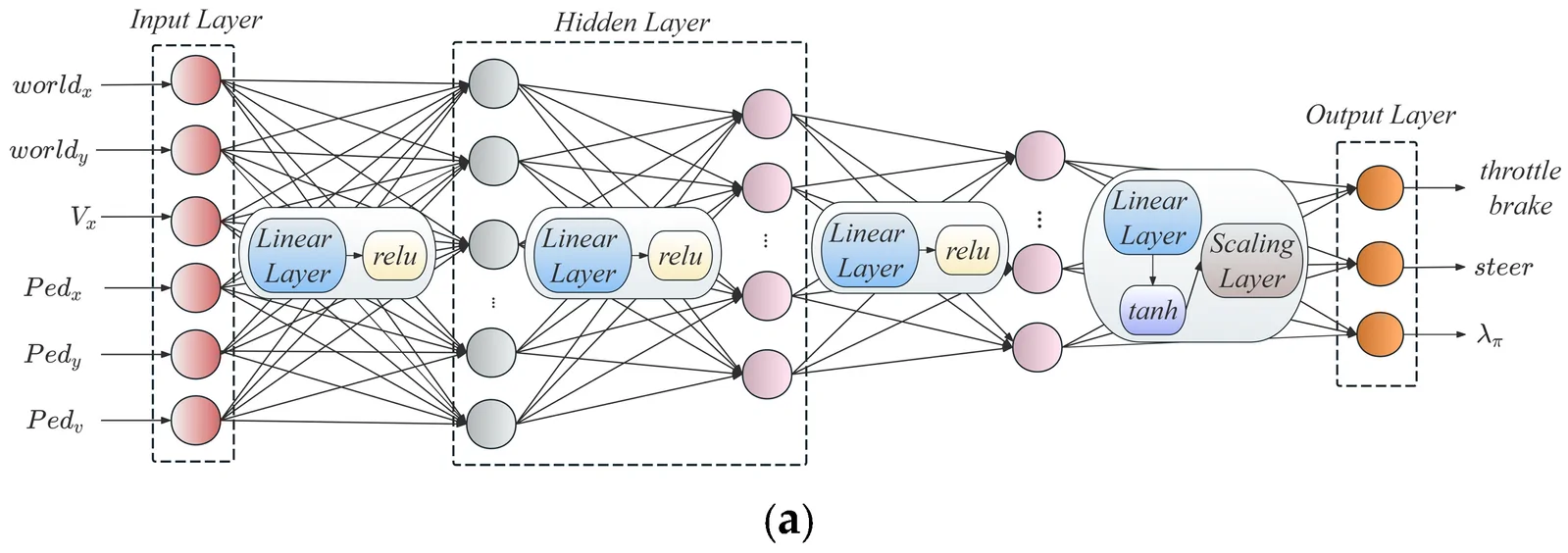

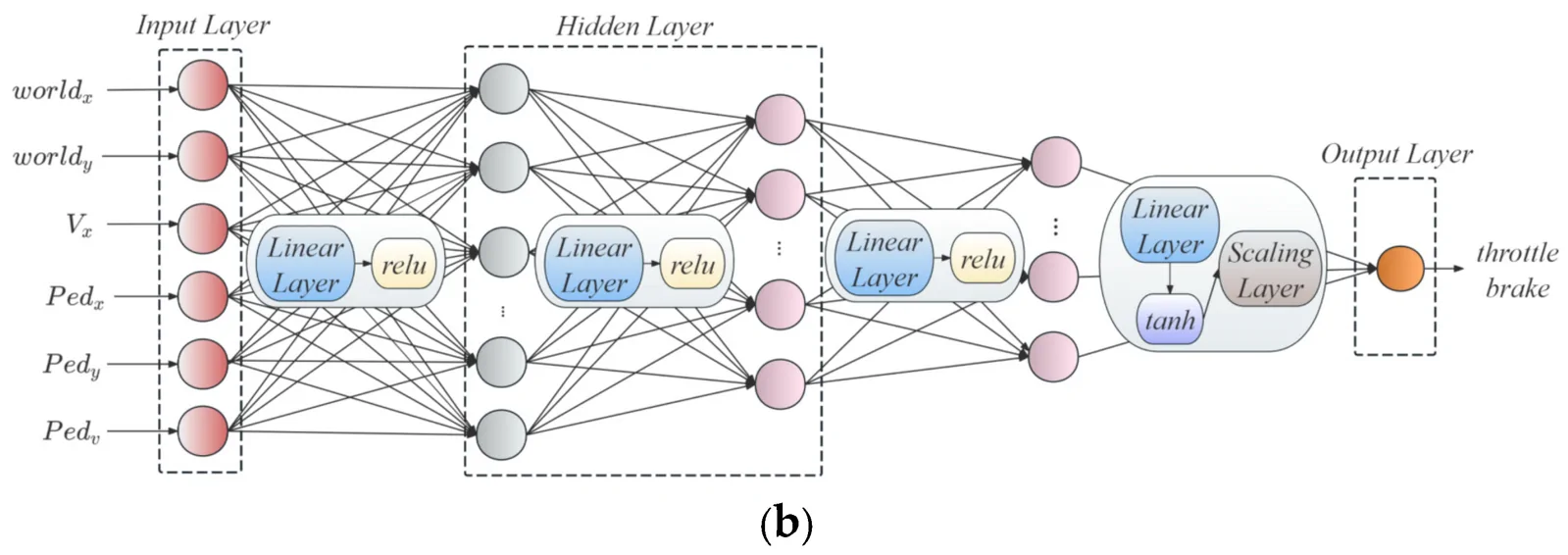
Actor network architecture: (**a**) DDPG-HP; (**b**) DDPG-RRT*.

**Figure 4:**
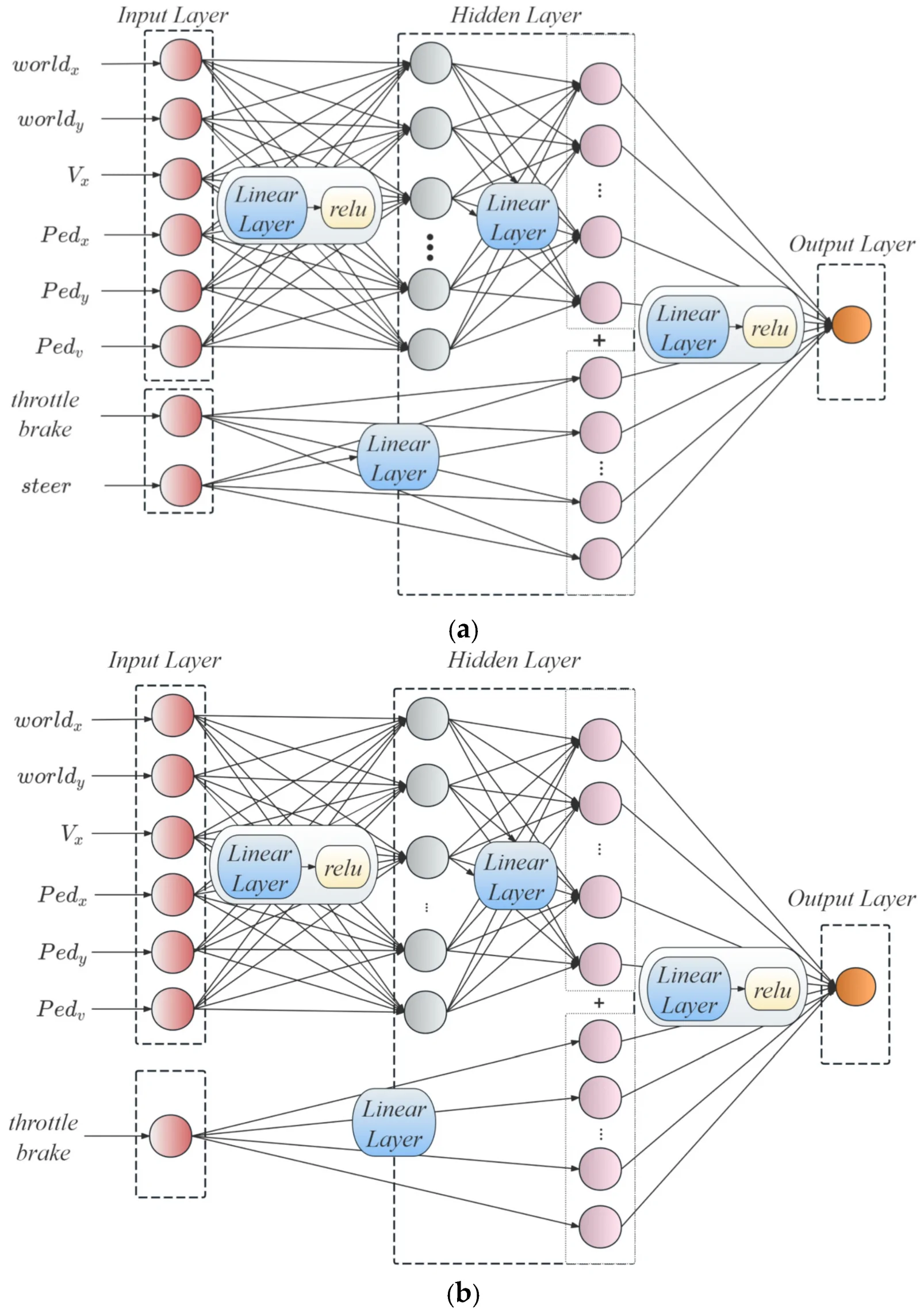
Critic network architecture: (**a**) DDPG-HP; (**b**) DDPG-RRT*.

To efficiently apply the DDPG algorithm for the coordinated lateral and longitudinal control of intelligent connected vehicles, this study addresses the real-time requirements of autonomous driving tasks by adopting the Actor–Critic dual-network architecture as the algorithmic core. Through interactive learning, the framework achieves an effective balance and collaborative optimization between control performance and safety constraints, as illustrated in Figure 3 and Figure 4. The network design considers the real-time demands of autonomous driving tasks, balancing the number of layers and neurons to strike a balance between computational efficiency and expressive power.

The Actor network serves as a policy approximator π*_ϕ_*(*S*). Its input includes the current environmental state *S*, which comprises vehicle states, obstacle information, and road features. The output consists of continuous control actions a = [*δ*, throttle, brake], where *δ* is the steering angle and throttle and brake represent longitudinal control commands. The network parameters *ϕ* are updated via policy gradient methods to maximize the expected cumulative reward. The Actor network adopts a structure of “Input layer → Hidden layer 1 (Linear + ReLU) → Hidden layer 2 (Linear + ReLU) → Output layer (Linear + Tanh).” The hidden layers contain 256 and 128 neurons, respectively. The output layer uses the Tanh activation function to confine actions to the [−1, 1] interval, which is then mapped to the actual physical control range. This approach avoids the quantization error inherent in traditional discrete-action methods, thereby enabling smoother trajectory-tracking capabilities.

To evaluate the integrated performance of the DDPG-HP algorithm, this paper introduces an additional comparative scenario: a hierarchical control architecture that decouples lateral path planning from longitudinal speed control. The upper layer employs an improved RRT* algorithm for real-time path planning, and the lower layer uses the DDPG algorithm as a longitudinal controller, which autonomously learns to optimize speed-tracking strategies, thereby guaranteeing both driving safety and passenger comfort in dynamic scenarios such as pedestrian crossings. The detailed structure of the Actor network is shown in Figure 3.

The Critic network is responsible for evaluating the value of state-action pairs *Q_θ_*(*s*, *a*). It takes the state *s* and action *a* as inputs and outputs the action-value *Q*-value to assess the long-term return of taking the specified action *a* in state *s*. 128 samples are collected from the experience replay buffer: (*s*, *a*, *r*, *s*′), and the target *Q*-value is calculated:

(2) $$y=r(s,a)+\gamma Q_{\theta^{\prime }}(s^{\prime },\pi_{\phi^{\prime }}(s^{\prime }))$$

where *r*(*s*, *a*) is the immediate reward, *γ* is the discount factor, $Q_{\theta^{\prime }}$ is the target Critic network, and $\pi_{\phi^{\prime }}$ is the target Actor network. The parameters of the target networks, *θ*′ and *ϕ*, are slowly tracked to the online network parameters *θ* and *ϕ*′ via a soft update strategy. The optimization objective of the Critic network is to minimize the mean squared error *ℒ*(*θ*) between the predicted *Q*-value and the target *Q*-value:

(3) $$L(\theta )=E_{(s,a,r,s^{\prime })\sim D}[{\left(Q_{\theta }(s,a)-y\right)}^{2}]$$

where *D* represents the experience replay buffer. The Adam optimizer is used, incorporating regularization and gradient clipping to ensure training stability. The agent collects experience data by interacting with the environment and updates the Actor network parameters *ϕ* using the deterministic policy gradient:

(4) $$\nabla_{\phi }J(\phi )=E_{s\sim D}[\nabla_{a}Q_{\theta }(s,a){|}_{a=\pi_{\phi }(s)}\nabla_{\phi }\pi_{\phi }(s)]$$

To break the correlation between value estimation and policy updates during training, a target network mechanism is established. This includes the Target–Actor network $\pi_{\phi^{\prime }}$ and the Target–Critic network, which share the same architecture as the online networks. The target network parameters are slowly tracked to the online network parameters using a soft update strategy: *ϕ*′ ← *τϕ* + (1 − *τ*)*ϕ*′, *θ*′ ← *τθ* + (1 − *τ*)*θ*′. Here, *τ* is the update coefficient, set to 0.003.

The network architecture also consists of two hidden layers with 256 and 128 neurons, respectively, using ReLU as the activation function. The final *Q*-value estimate is output through a linear layer, as shown in Figure 4.

Furthermore, this study incorporates Ornstein–Uhlenbeck (OU) noise into the algorithm to enhance the model’s exploratory capability in the environment. Concurrently, an experience replay mechanism is employed to store state transition samples generated from the agent’s interaction with the environment. An off-policy method is utilized to train the neural network in batches, thereby improving data utilization efficiency and enhancing the stability of the learning process.

### 3.4. Driving Tendency Network Design

#### Driving Tendency Network

In complex and dense urban road environments, human drivers typically rely on experiential intuition for real-time responses rather than directly planning high-level behaviors and executing precise control. The autonomous driving task can be viewed as a search and optimization problem within a global policy space [17]. The driving tendency mechanism is introduced to constrain the policy search space, improve learning efficiency, and enhance decision-making rationality by integrating prior knowledge from human driving experience. This enables the intelligent agent to efficiently converge towards feasible driving strategies that align with human habits while ensuring safety. Interaction models based on mechanisms are applied to determine appropriate initial values for optimization. Analogous to the decision-making characteristics of human driving, “driving tendency” is defined to quantify decision-making preferences in driving behavior. The driving tendency network continuously optimizes its outputs to provide reasonable driving guidance for the system, effectively constraining the direction of policy search. Its outputs, together with those of the interaction model, serve as inputs to a nonlinear cost function. Solving this optimization problem generates safe and reliable longitudinal and lateral control commands.

Actor–Critic, as a typical reinforcement learning framework, aims for the Actor to maximize cumulative rewards. Under the premise of a well-designed reward function, the optimization objective of the driving tendency network aligns with that of the Actor: to learn safe and efficient driving behaviors by maximizing long-term returns, ultimately achieving or surpassing human-level driving performance. Equation (5) is defined below:

(5) $$s_{t}={\left[x_{car},y_{car},v_{car},{\dot{\varphi }}_{car},{\left\{obs_{i}\right\}}_{i=1}^{N}\right]}^{T}$$

where ${\dot{\varphi }}_{\mathrm{car}}$ represents the vehicle’s yaw rate, and obs*_i_* denotes the information of obstacles. The driving tendencies output by the network include lane keeping, lane changing, and risk aversion, defined as follows:

(6) $$\lambda_{t}={\left[\lambda_{\mathrm{agg}},\lambda_{\mathrm{cons}},\lambda_{\mathrm{LC}},\lambda_{\mathrm{risk}}\right]}^{T}$$

The driving-tendency coefficients *λ_t_* are generated jointly by the Actor and Critic networks. The Actor network outputs both the control action *a* and the tendency vector *λ_π_*, while the Critic network outputs both the state-action value *Q* and the tendency vector *λ_Q_*:

(7) $$\left[a,\lambda_{\pi }]\;=\pi_{\phi }(s\right)$$

(8) $$\left[Q,\lambda_{Q}]=Q_{\theta }(s,a\right)$$

where *λ_π_* = [*λ*_*π*,*agg*_, *λ*_*π*,*cons*_, *λ*_*π*,*LC*_, *λ*_*π*,*risk*_]*^T^* and *λ_Q_* = [*λ*_*Q*,*agg*_, *λ*_*Q*,*cons*_, *λ*_*Q*,*LC*_, *λ*_*Q*,*risk*_]*^T^* are constrained to (0, 1) by a Sigmoid activation at the output layer. The tendency vector used for reward shaping is taken from the Actor network, i.e., *λ_t_* = *λ_π_*.

To ensure consistency between the two networks, a consistency loss is defined as

(9) $$L_{\mathrm{consist}}(\theta ,\phi )=E_{(s,a,r,s^{\prime })\sim D}\left[{\left\|\lambda_{\pi }-\lambda_{Q}\right\|}_{2}^{2}\right]$$

The TD loss of the Critic is

(10) $$L_{TD}(\theta )=E_{(s,a,r,s^{\prime })\sim D}\left[{\left(Q_{\theta }(s,a)-y\right)}^{2}\right]$$

The total loss is

(11) $$L_{TD}(\theta )=E_{(s,a,r,s^{\prime })\sim D}\left[{\left(Q_{\theta }(s,a)-y\right)}^{2}\right]$$

where *a* is the weight of the consistency loss. By minimizing the total loss, the generation of *λ_t_* is optimized jointly with the Actor–Critic parameters, without manual labels.

(1) Collision Risk Assessment and Grading

(12) $$R_{\mathrm{collision}}=I\left(\underset{i}{\min}\sqrt{{\left(x_{\mathrm{car}}-x_{i}\right)}^{2}+{\left(y_{\mathrm{car}}-y_{i}\right)}^{2}}<d_{\mathrm{safe}}\right)$$

where I is the indicator function, serving as a flag for collision avoidance lane change. The safe distance is defined as

(13) $${\mathrm{Flag}}_{d}=\left\{\begin{array}{cc} 1, & \underset{i}{\min}\sqrt{{\left(x_{\mathrm{car}}-x_{i}\right)}^{2}+{\left(y_{\mathrm{car}}-y_{i}\right)}^{2}}<10\\ 0, & t_{{\mathrm{ttc}}_{i}}<5.0\\ -1, & \mathrm{otherwise} \end{array}\right.$$

(14) $${\mathrm{Flag}}_{p}=\left\{\begin{array}{cc} 1, & t_{{\mathrm{ttc}}_{p}}<t_{\mathrm{premin}}+0.3\\ 0, & t_{\mathrm{premin}}+0.3\leq t_{{\mathrm{ttc}}_{p}}<t_{\mathrm{premin}}+1.0\\ -1, & y_{p}>3.0\;\mathrm{or}\;y_{p}<-7.0 \end{array}\right.$$

where $t_{{\mathrm{ttc}}_{i}}=\frac{x_{i}-x_{\mathrm{car}}}{v_{\mathrm{car}}-v_{i}}$ is the time to collision between the vehicle and the *i*-th obstacle, categorized as danger, warning, and safe, respectively [18]. Vehicles with potential impact are defined as the following set: ℝ⊆ℚ, where $\mathbb{R}=[x_{obs1},y_{obs1},v_{obs1},\ldots ,x_{obsn},y_{obsn},v_{obsn}]$.

(15) $$t_{P_{\mathrm{remain}}}=\max\left(0,\frac{3-y_{p}}{\max(v_{\mathrm{ped}},0.01)}\right)$$

where $t_{P_{\mathrm{remain}}}$ is the remaining time for the pedestrian to cross the road, and $t_{{\mathrm{ttc}}_{p}}=\frac{130-x_{\mathrm{car}}}{\max(v_{x},0.01)}$ is the time to potential collision between the vehicle and the pedestrian.

(2) Lane Change Decision

The lane centerline positions are *y*_lane_ ∈ [−5.25, −1.75, 1.75], corresponding to the three lanes, respectively. The lane change flag is defined as

(16) $${\mathrm{Flag}}_{c}=\left\{\begin{array}{cc} 1, & \exists |y_{\mathrm{car}}-y_{i}|\leq 1.0\;\mathrm{and}\;|x_{\mathrm{car}}-x_{i}|<d_{\mathrm{safe}}\\ 0, & \mathrm{otherwise} \end{array}\right.$$

where *d*_safe_ = min(15, 6 + 1.8 × *v*_car_) is the dynamic safe distance.

### 3.5. DDPG-HP Reward Function Design

In deep reinforcement learning for autonomous driving policy learning, the reward function serves as a critical mechanism to guide the agent’s learning. To align human–machine collaborative decision-making with natural human driving preferences, this paper decomposes the reward function into four components: driving tendency reward, process optimization reward, safety assurance reward, and task completion reward. A dense reward design incorporates multi-dimensional sub-items and an adaptive weighting mechanism based on driving tendencies. This guides the agent to gradually develop decision-making strategies that conform to human driving habits during training. The overall reward function is designed as follows:

(17) $$r_{t}=r_{\mathrm{HP}}+r_{\mathrm{progress}}+r_{\mathrm{safety}}+r_{\mathrm{completion}}$$

#### 3.5.1. Design of the Reward Function for the Driving Tendency Network

Human drivers typically prioritize safety, pursue smoothness and comfort, and aim for efficient passage when safety conditions allow. To reflect these tendencies, this paper categorizes the reward into three main types: lane keeping, lane changing, and risk aversion. The learned driving-tendency outputs are transformed into weighting factors for each reward sub-item, enabling adaptive weighting rather than static predefined weights. The reward function is designed as follows.

(1) Lane Keeping Reward

To encourage the vehicle to stay near the center of the lane under collision-free conditions (Flag_d_ = −1), the reward is formulated as $r_{\mathrm{lane}}=-\alpha_{\mathrm{lane}}(\lambda_{\mathrm{cons}})\cdot {\left\|y_{\mathrm{car}}-y_{\mathrm{center}}\right\|}^{2}$.

(2) Lane Change Reward

When a collision risk is detected, the vehicle is incentivized to change lanes *y_t_*:

(18) $$r_{\mathrm{LC}}=\left\{\begin{array}{cc} (0.5+0.5\lambda_{\mathrm{LC}})\cdot (50-2|y_{t}-y_{\mathrm{car}}{|}^{-1}), & {\mathrm{Flag}}_{c}=1\\ 0, & \mathrm{otherwise} \end{array}\right.$$

(3) Risk Aversion Reward

Punishments are applied when the vehicle approaches obstacles, using a dynamic safe following distance. In dangerous states, inverse-distance penalties and negative speed rewards are imposed for vehicles that are laterally close and longitudinally hazardous, encouraging deceleration or lane changes:

(19) $$r_{\mathrm{risk}}=-(0.3+0.7\cdot \lambda_{\mathrm{risk}})\cdot \sum_{i=1}^{N}\left(\frac{2.0}{d_{i}}+\frac{0.5}{d_{i}^{2}}\right)$$

where $d_{i}=\sqrt{{\left(x_{\mathrm{car}}-x_{i}\right)}^{2}+{\left(y_{\mathrm{car}}-y_{i}\right)}^{2}}$, so the total reward is *r*_HP_ = *r*_lane_ + *r*_LC_ + *r*_risk_.

#### 3.5.2. Process Optimization Reward

To continuously optimize behaviors during the driving process, reflecting the daily human driver inclinations towards comfort, stability, speed reasonableness, and forward progress, the process reward is designed as

(20) $$r_{\mathrm{process}}=r_{\mathrm{progress}}+r_{\mathrm{comf}}+r_{\mathrm{lane}}+r_{\mathrm{yaw}}+r_{\mathrm{changelane}}+r_{v}$$

(1) Progress Reward, encouraging continuous advancement: $r_{\mathrm{progress}}=0.8x_{\mathrm{car}}$.
(2) Comfort Reward, constructing multi-dimensional penalties based on human comfort thresholds:
  (21) $$r_{\mathrm{comf}}=-0.1\cdot k{\left(a_{x}-a_{\mathrm{comf}}\right)}^{2}-0.08a_{x}^{2}-0.018j_{x}^{2}-0.040{\dot{\varphi }}_{\mathrm{car}}^{2}-0.025{\ddot{\varphi }}_{\mathrm{car}}^{2}$$

(3) Lane Keeping Reward

(22) $$r_{\mathrm{lane}}=\left\{\begin{array}{cc} -0.5\cdot d_{\mathrm{lane}}^{2}, & {\mathrm{Flag}}_{c}=0\\ -0.1\cdot d_{\mathrm{lane}}^{2}, & {\mathrm{Flag}}_{c}=1 \end{array}\right.$$

where $d_{\mathrm{lane}}=\underset{i}{\min}|y_{\mathrm{car}}-y_{\mathrm{lane},i}|$, *y*_lane_ = {−5.25, ±1.75}.

(4) Yaw Control Reward During Lane Change
  The yaw control reward during lane change *r*_yaw_ and successful lane change reward *r*_changelane_ enabling smooth lane changes:
  (23) $${\dot{\varphi }}_{\mathrm{des}}=\mathrm{clip}\left(95\cdot \frac{y_{\mathrm{target}}-y_{\mathrm{car}}}{\max(v_{\mathrm{car}},10)},\pm 35\right)$$
  Therefore, set $r_{\mathrm{yaw}}=-2.0|\varphi_{\mathrm{car}}-\varphi_{\mathrm{des}}|$.
(5) Speed Planning Reward

**Figure 5:**
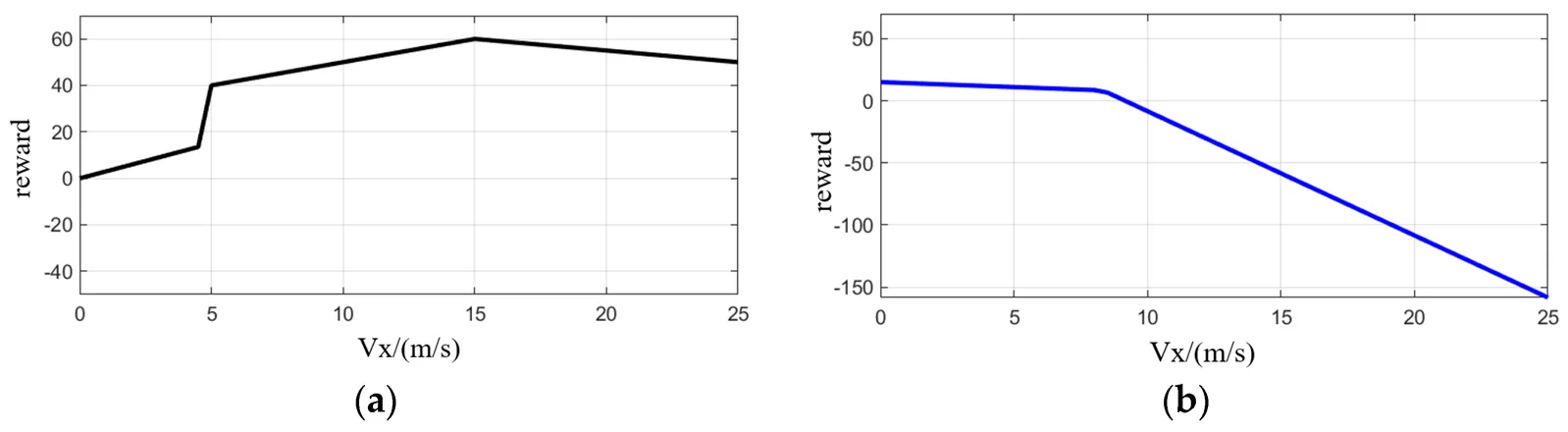
Velocity efficiency reward in different scenarios: (**a**) normal road section; (**b**) pedestrian crossing section.

The speed planning reward *r_v_* is designed with a segmented speed curve. The vehicle receives segmented rewards in normal sections, and is encouraged to decelerate in crosswalk areas to reflect human speed preferences in different scenarios, as shown in Figure 5.

#### 3.5.3. Safety Reward

Centered on the human tendencies of ‘zero tolerance for risk’ and ‘courteous compliance’, this component primarily penalizes potential or actual hazardous behaviors, mainly including the following sub-items: *r*_safe_ = *r*_ped_ + *r*_coll_.

(1) Reward for Slowing Down for Obstacle Vehicles and Yielding to Pedestrians

Based on the graded speed reward determined by the *TTC* with obstacle vehicles and the pedestrian’s remaining crossing time, this encourages the vehicle to safely yield to pedestrians:

(24) $$r_{\mathrm{ped}}=\left\{\begin{array}{cc} -\frac{3.6\times v_{x}}{60}, & {\mathrm{Flag}}_{d}=1\;∥\;{\mathrm{Flag}}_{p}=1\\ 0, & {\mathrm{Flag}}_{d}=0\;∥\;{\mathrm{Flag}}_{p}=0\\ \frac{3.6\times v_{x}}{60}, & {\mathrm{Flag}}_{d}=-1\;∥\;{\mathrm{Flag}}_{p}=-1 \end{array}\right.$$

(2) Collision Termination Penalty

A heavy penalty *r*_coll_ = −100 is imposed when a collision with a vehicle or pedestrian occurs, or when boundary violations happen.

#### 3.5.4. Completion Reward

This includes the task completion reward and the global efficiency reward, defined as *r*_completion_ = *r*_reach_ + *r*_eff_.

(1) Task Completion Reward: $r_{\mathrm{reach}}=\left\{\begin{array}{cc} 2000, & x_{\mathrm{car}}>130\\ 0, & \mathrm{otherwise} \end{array}\right.$
(2) Global Efficiency Reward: $r_{\mathrm{eff}}=r_{{\mathrm{eff}}_{\mathrm{goal}}}+r_{{\mathrm{eff}}_{\mathrm{dist}}}$:

$r_{{\mathrm{eff}}_{\mathrm{goal}}}=\left\{\begin{array}{cc} 500, & \mathrm{cars}\;\mathrm{and}\;\mathrm{pedestrians}\;\mathrm{crossed}\;\mathrm{line}\\ 0, & \mathrm{otherwise} \end{array}\right.$, $r_{{\mathrm{eff}}_{\mathrm{dist}}}=\frac{5\times v_{\mathrm{avg}}\times 3.6}{v_{\mathrm{car}}}-10$ where $v_{\mathrm{avg}}=\frac{x_{\mathrm{car}}-x_{0}}{\mathrm{timestamp}+0.02},\;\;\;\;x_{0}=30$.

### 3.6. V2X Communication

**Figure 6:**
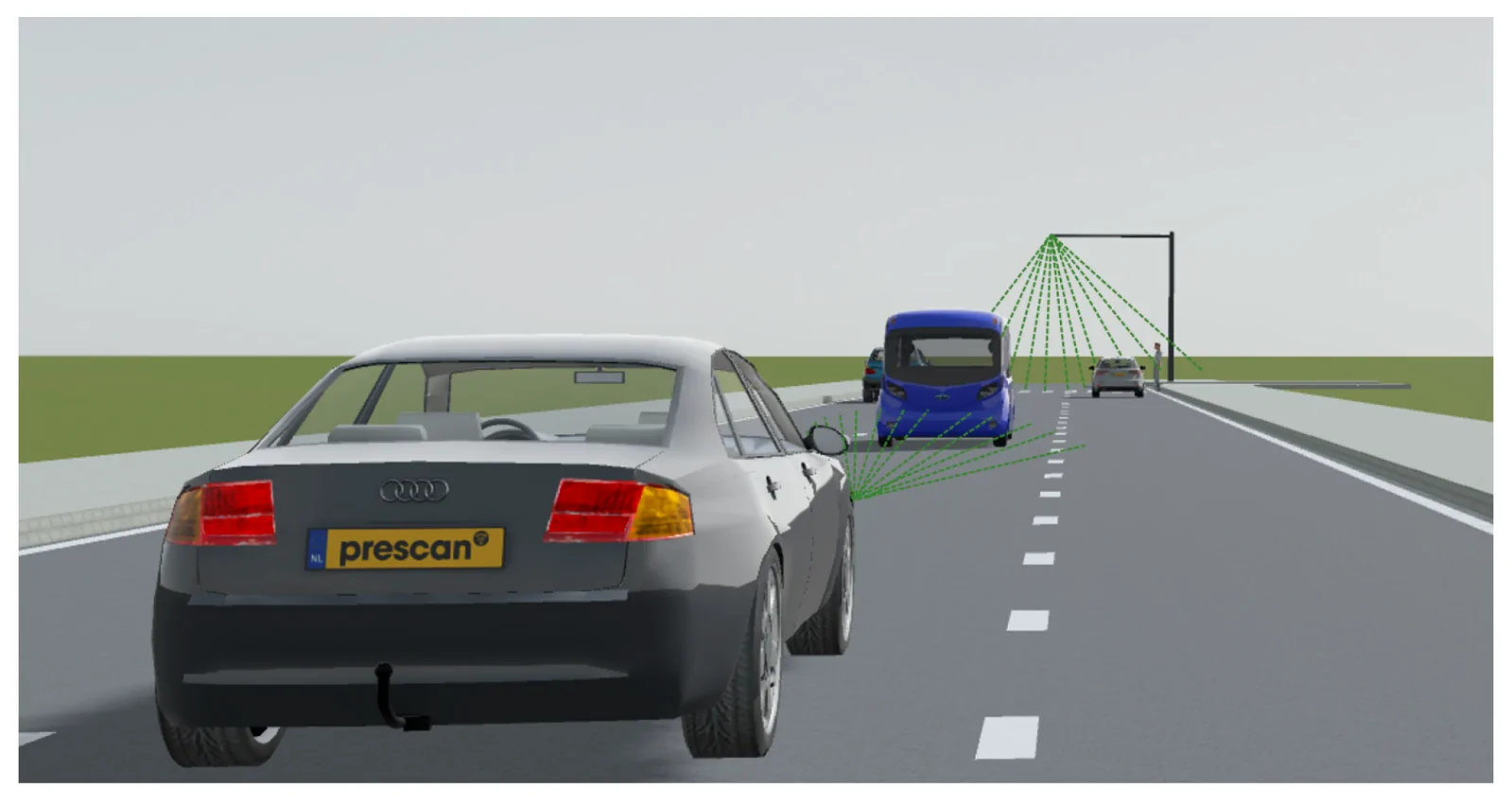
V2X communication diagram. The green dashed lines represent the sensing beam of the on-board sensor.

During V2X communication and sensor data transmission, packet loss and delay frequently occur, leading to abrupt changes or missing input states. This directly impacts the continuity of control decisions and the stability of the training process [19]. To address this problem, this paper designs a perception signal compensation mechanism that combines a historical buffer and Kalman filtering. When packet loss or delay is detected, historical state sequences are utilized to predict and smooth the current state, thereby providing the reinforcement learning agent with more reliable and continuous observation inputs, as illustrated in Figure 6. This design mimics the human driver’s “inertial perception” of vehicle states—the ability to estimate the current motion state based on experience even when precise feedback is temporarily unavailable. The inputs to the signal filtering module include the current time and raw perception states, including *x*_car_, *y*_car_, *v*_car_, *a*_car_, Flag_loss_ and *Delay*_num_. The processing workflow is divided into three stages: delayed data processing, anomaly detection and state compensation, and state updating.

The V2X communication anomaly model adopted in this paper is based on distance-dependent packet loss probability and Poisson-distributed delays. This simplified model captures the core characteristics of signal loss and delay. It does not account for correlated packet losses, multipath fading, sudden network congestion in dense traffic, or cyber-physical security threats, which may affect the Kalman-filter compensation performance in different ways. The focus of this paper is on the compensation algorithm’s response to packet loss and delay anomalies, rather than on reproducing every detail of real-world V2X environments. Therefore, the simplified model is used as a basis for validating the compensation mechanism, while the above factors remain to be addressed in future work.

#### 3.6.1. Packet Loss and Delay Module

This paper designs a parameterized packet loss and delay simulation module. It generates random packet loss events and Poisson-distributed [20] delays through a probability model. When packet loss occurs, all state inputs are set to zero, providing representative anomalous inputs for the subsequent signal filtering module. Based on a signal packet loss probability model dependent to communication distance, it simulates the real-world characteristic of deteriorating communication quality over long distances. The formulas for packet loss probability calculation and packet loss judgment are as follows:

(25) $$Q=\frac{P\cdot {\left(x_{\mathrm{car}}+\Delta t\right)}^{-\phi }}{P\cdot {\left(x_{\mathrm{car}}+\Delta t\right)}^{-\phi }+O}$$

where Δ*t* is the additional distance offset; *P* is the scale parameter, set to 10; *φ* is the distance decay exponent, set to 0.5; set Flag_loss_ = (*Q* < *β*), where *β* is the packet loss threshold, set to 0.82; *O*~N(*μ* = 0, *σ* = 0.2) is a Gaussian noise term introduced to add random fluctuations and avoid deterministic packet loss. Transmission delay is simulated using a Poisson distribution to capture its randomness: Delay_num_ = min(poisson(1),5).

#### 3.6.2. Kalman Filter Prediction and Compensation Model

When packet loss or delay is detected, a Kalman filter with reverse smoothing is employed. Using a historical observation data buffer, it provides an optimal estimation of the vehicle’s motion state during communication anomalies, ensuring the continuity and safety of the autonomous driving system.

(1) The state prediction ${\hat{x}}_{k}^{-}$ is ${\hat{x}}_{k}^{-}=Ax_{k-1}$
(2) The prediction covariance matrix $P_{k}^{-}$ is $P_{k}^{-}=AP_{k-1}A^{T}+Q$
(3) The Kalman gain $K_{k}$ is $K_{k}=P_{k}^{-}H^{T}{\left(HP_{k}^{-}H^{T}+R\right)}^{-1}$
(4) The optimal state estimate ${\hat{x}}_{k}$ is ${\hat{x}}_{k}={\hat{x}}_{k}^{-}+K_{k}\left(z_{k}-H{\hat{x}}_{k}^{-}\right)$
(5) The updated state estimate covariance is $P_{k}=\left(I-K_{k}H\right)P_{k}^{-}$

where *x_k_* is the state vector, *z_k_* is the high-definition map measured state, ***A*** is the state transition matrix, ***H*** is the state observation matrix, the process noise covariance matrix ***Q*** and the observation noise covariance matrix ***R*** are defined as $x_{k}={\left[\begin{array}{ccc} x_{{\mathrm{car}}_{k}} & v_{k} & a_{k} \end{array}\right]}^{T},\;z_{k}={\left[\begin{array}{cc} {\tilde{x}}_{{\mathrm{car}}_{k}} & {\tilde{v}}_{k} \end{array}\right]}^{T}$, where ${\tilde{x}}_{{\mathrm{car}}_{k}}$ and ${\tilde{v}}_{k}$ are noisy observation values.

$$A=\left[\begin{array}{ccc} 1 & \Delta t & \frac{1}{2}\Delta t^{2}\\ 0 & 1 & \Delta t\\ 0 & 0 & 1 \end{array}\right],\;H=\left[\begin{array}{ccc} 1 & 0 & 0\\ 0 & 1 & 0 \end{array}\right],\;Q=\left[\begin{array}{ccc} 5 & 0 & 0\\ 0 & 0.5 & 0\\ 0 & 0 & 0.1 \end{array}\right],\;R=\left[\begin{array}{cc} 1 & 0\\ 0 & 0.5 \end{array}\right]$$

Among them, $x_{{\mathrm{car}}_{k}}$, $v_{k}$ and $a_{k}$ represent the longitudinal position, velocity, and acceleration at step *k*, respectively. Using the latest valid historical data from the buffer as the initial state and covariance, a prediction and update cycle is performed frame by frame from the end of the buffer forward. This process outputs a smoothed estimate for the current time step, effectively suppressing random fluctuations in observation noise while maintaining the system’s ability to track dynamics in real time.

## 4. Simulation Verification and Analysis

**Figure 7:**
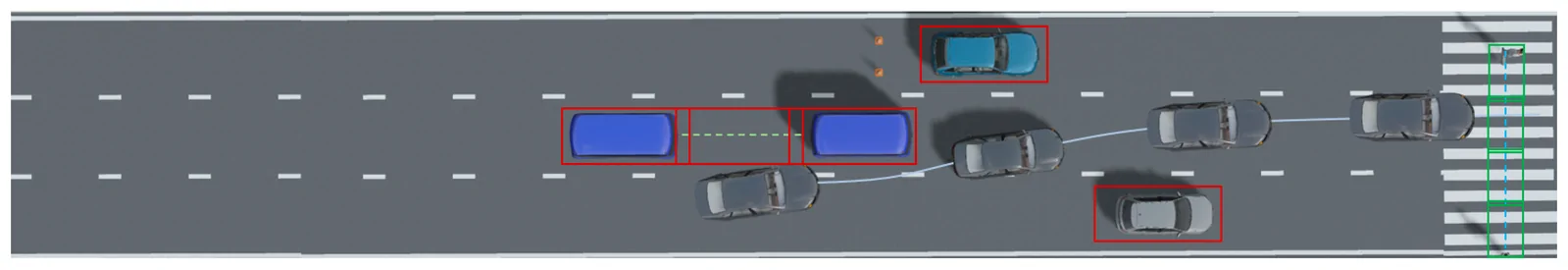
Schematic of the multi-vehicle dynamic avoidance scenario.

To validate the performance of the DDPG-HP control algorithm proposed in this study, a lateral control simulation model was developed based on a co-simulation platform integrating MATLAB/Simulink (version R2022b, MathWorks, Natick, MA, USA) and Prescan (version 2021.1.0, Siemens Digital Industries Software, Leuven, Belgium). The designed simulation training scenario, as illustrated in Figure 7, features a unidirectional three-lane road with each lane being 3.5 m wide. The scenario includes a pedestrian crossing at a crosswalk, a stationary vehicle in Lane 1 due to a malfunction, a vehicle traveling at a constant speed in Lane 2, and a temporarily parked vehicle in Lane 3, which corresponds to three obstacle vehicles and one pedestrian (*n* = 3 in Equation (1)). The ego vehicle incorporates a vehicle dynamics model, and a V2X communication environment was established to form a traffic flow model centered around this vehicle.

### 4.1. Simulation Training Scenario Design

**Figure 8:**
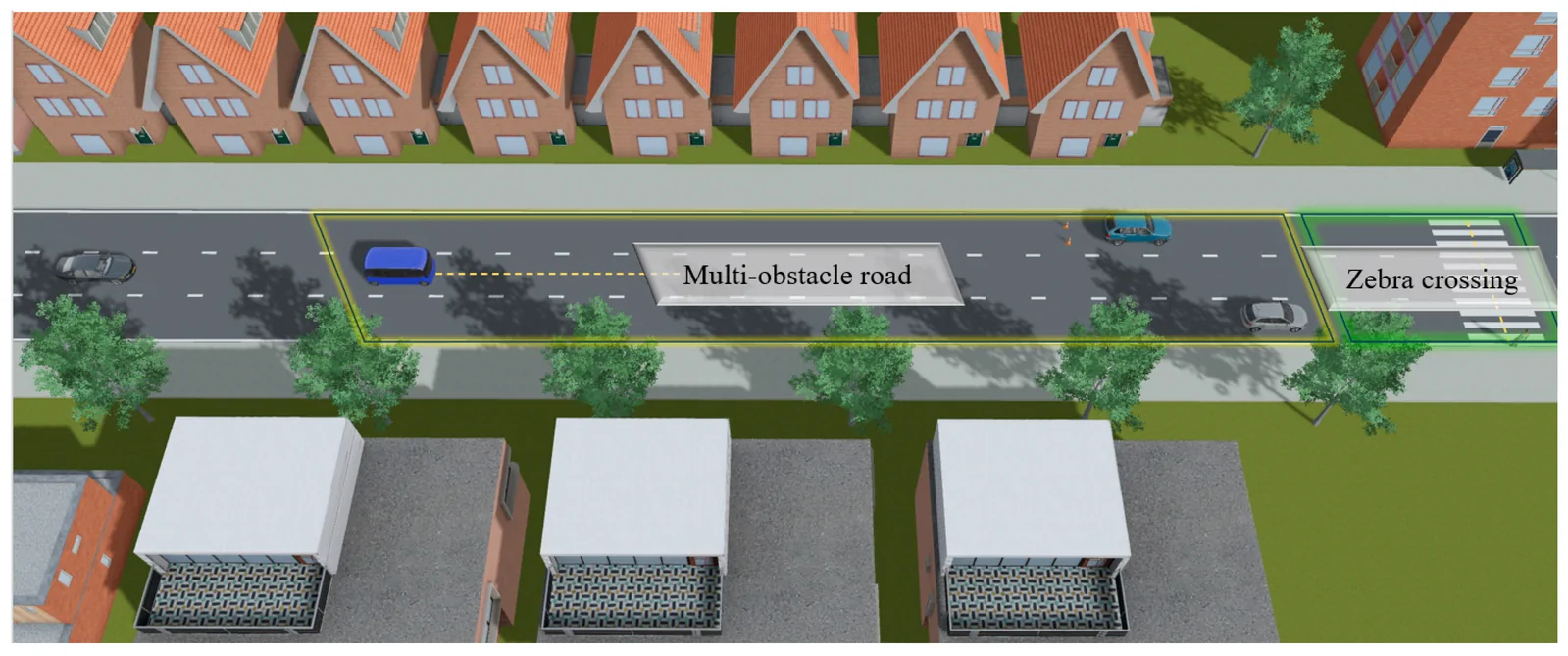
Training scene diagram.

The simulation training scenario was constructed based on the Prescan platform, as shown in Figure 8. This scenario is designed to include multi-obstacle road sections and crosswalk sections to simulate real-world urban traffic conditions.

### 4.2. V2X Communication Simulation Results

**Figure 9:**
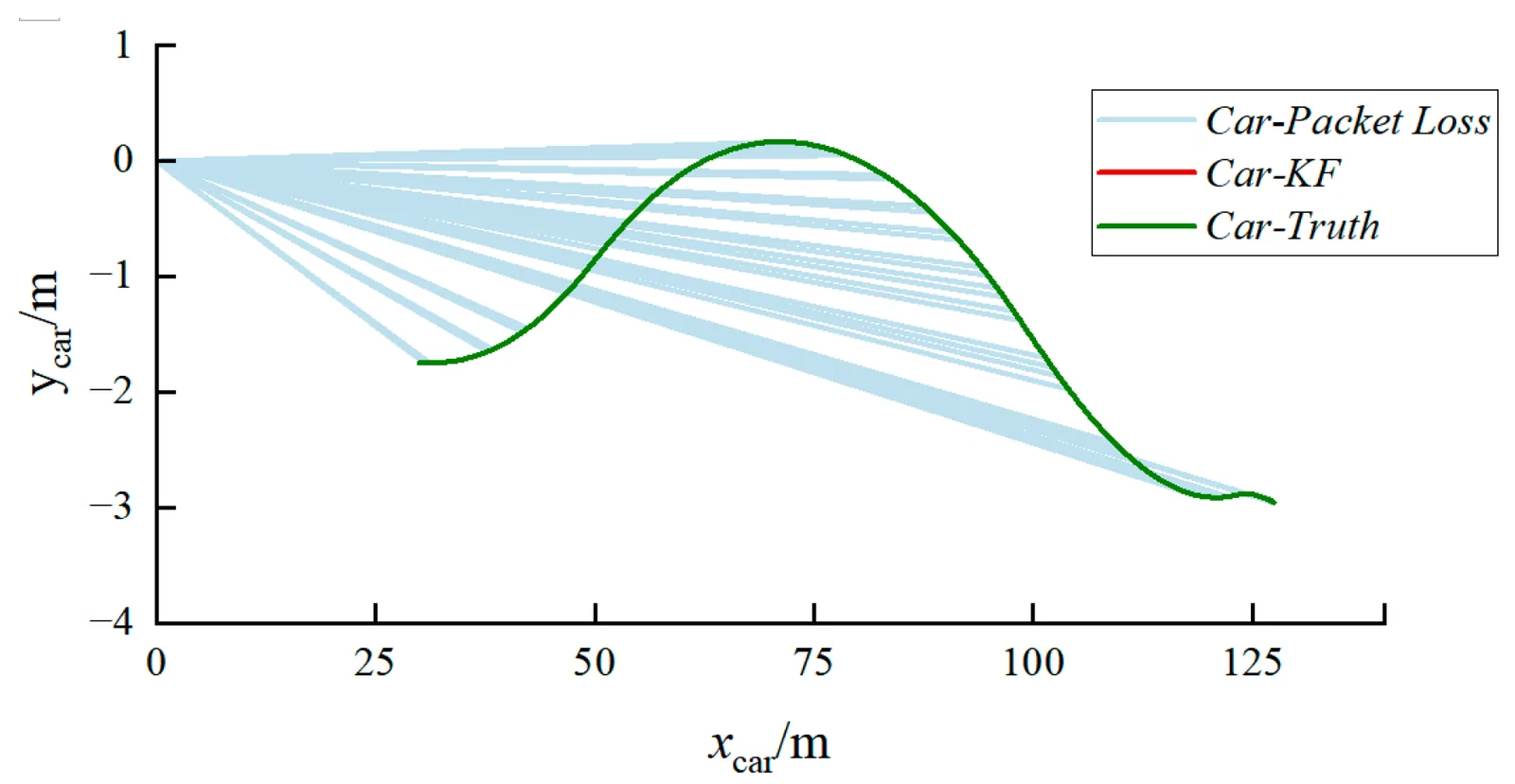
Comparison of vehicle trajectory compensation effects under V2X communication packet loss.

**Figure 10:**
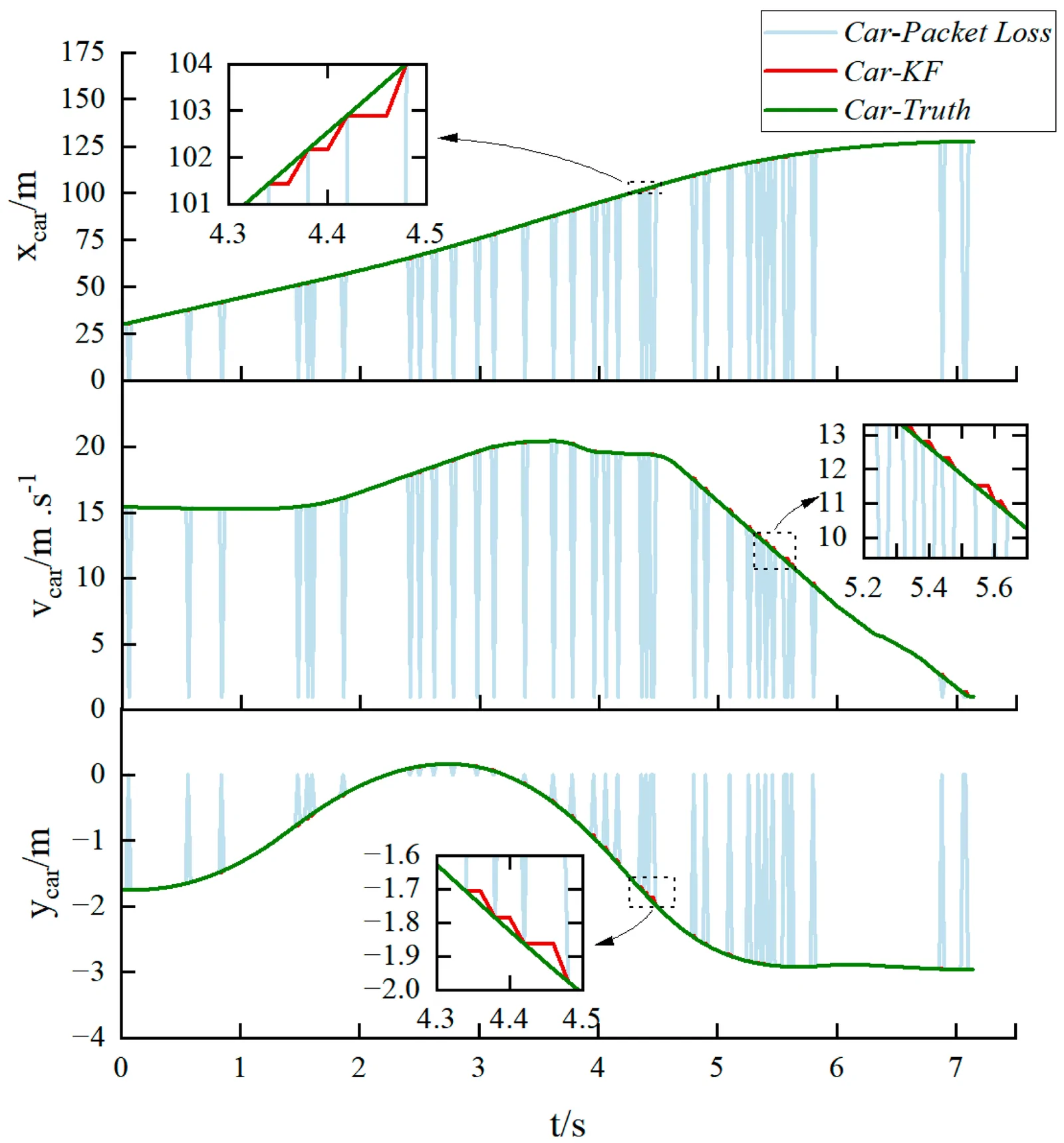
Comparison of longitudinal and lateral motion and velocity compensation effects under V2X communication packet loss.

To simulate the uncertainty of communication signal transmission in a vehicular network environment, this study designed a parameterized communication packet loss and delay simulation module. The module uses a packet loss probability model based on vehicle travel distance and a Poisson process to simulate signal delay characteristics. When packet loss occurs, the system sets the corresponding observation value to zero at the receiving end, simulating the impact of communication interruptions on the perception system. Figure 9 and Figure 10 compare packet loss occurrences, Kalman-filter state estimates, and actual vehicle motion states.

As shown in Figure 9 and Figure 10, within the blue vertical bar regions indicating intervals of communication packet loss or delay, uncompensated raw states exhibit abrupt changes or data gaps, leading to discontinuities or abnormal fluctuations in the vehicle’s position, velocity, and acceleration curves. After introducing the state compensation mechanism based on reverse smoothing Kalman filtering, the system can fully utilize historical motion trends and vehicle dynamics priors to perform smooth extrapolation and reconstruction of the state at the current moment. The compensated state curves show minimal error compared to the ideal continuous reference trajectory, significantly eliminating abnormal jumps in the original data and achieving continuous and stable estimation of the vehicle’s motion state.

The simulation results indicate that under abnormal communication conditions, Kalman filter compensation provides high-quality state inputs. These inputs effectively support the decision-making of the reinforcement learning controller. This enables the vehicle to maintain smooth driving trajectories and coherent control actions even in environments with partially missing observational information, thereby substantially enhancing the system’s adaptability and overall robustness in complex urban traffic scenarios and unstable V2X communication conditions.

### 4.3. Comparison of Different Learning Strategies

**Figure 11:**
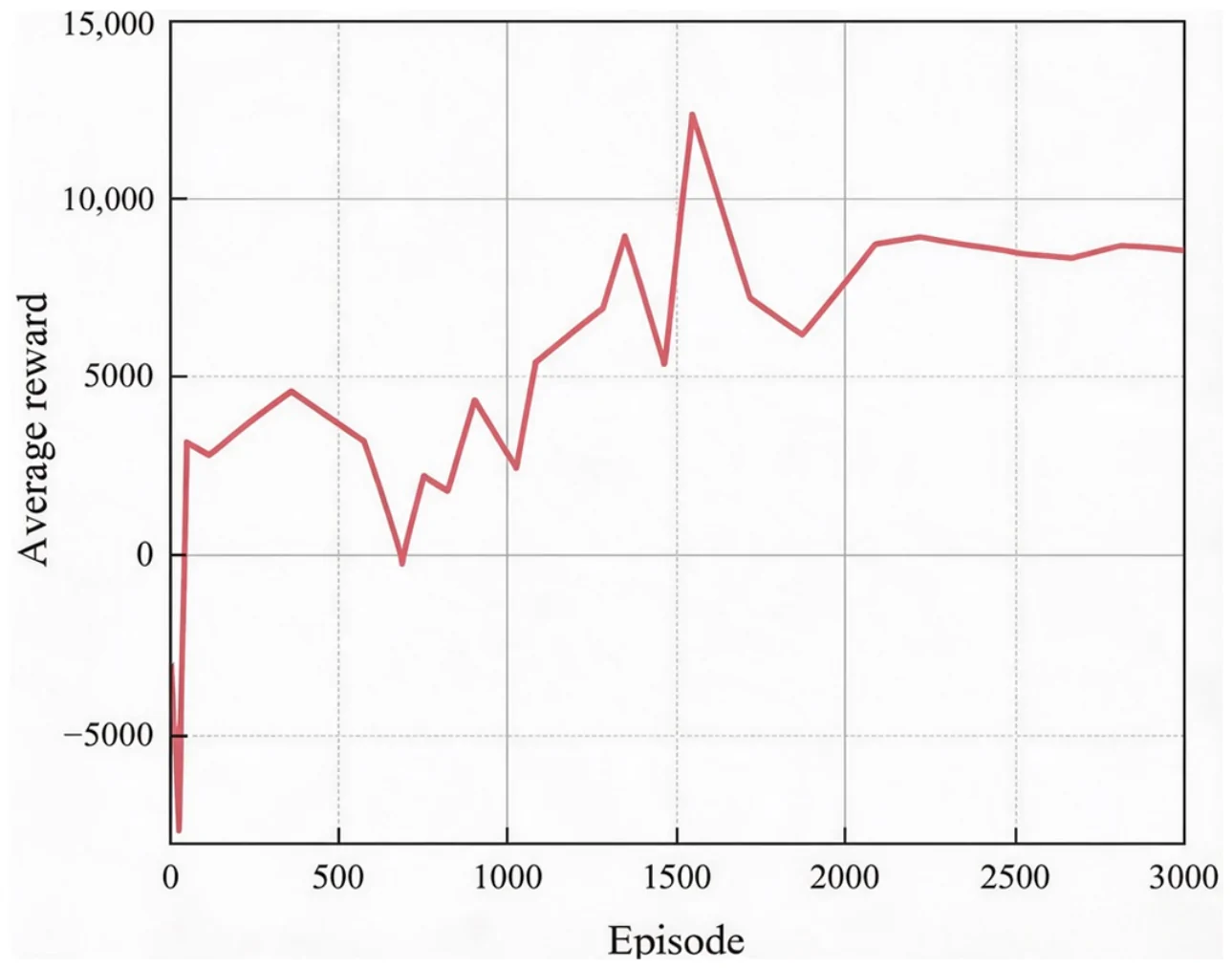
Average reward of DDPG-HP algorithm over 3000 episodes.

The training was terminated upon convergence, and Figure 11 presents the average reward curve over the actual 3000 training episodes. The curve transitioned from initial oscillations to gradual convergence and stabilization.

The DDPG-HP algorithm converges at around 3000 training episodes, whereas vanilla DDPG requires 6000 episodes. Under identical hardware conditions, the total training time of DDPG-HP is approximately 50% of that of vanilla DDPG, demonstrating that the human-driving-tendency prior reduces computational cost in the training phase.

For real-time performance evaluation within the simulation environment, we measured the inference latency of the Actor network on a desktop platform equipped with an AMD Ryzen AI 9 365 processor (2.00 GHz, designed and supplied by Advanced Micro Devices, Santa Clara, CA, USA) and 32 GB RAM. The average per-step inference latency is 0.13 ms (median: 0.11 ms), yielding a theoretical upper-bound inference rate of roughly 7600 FPS. The Actor network consumes only 0.12 MB of memory. Given the 20 ms control cycle of the simulation (*T_s_* = 0.02 s), the measured inference latency satisfies the real-time constraint of the simulation.

To verify the effectiveness of the DDPG-HP algorithm, this study established a controlled experiment, comparing the designed algorithm with the traditional DDPG method under the same simulation environment.

**Figure 12:**
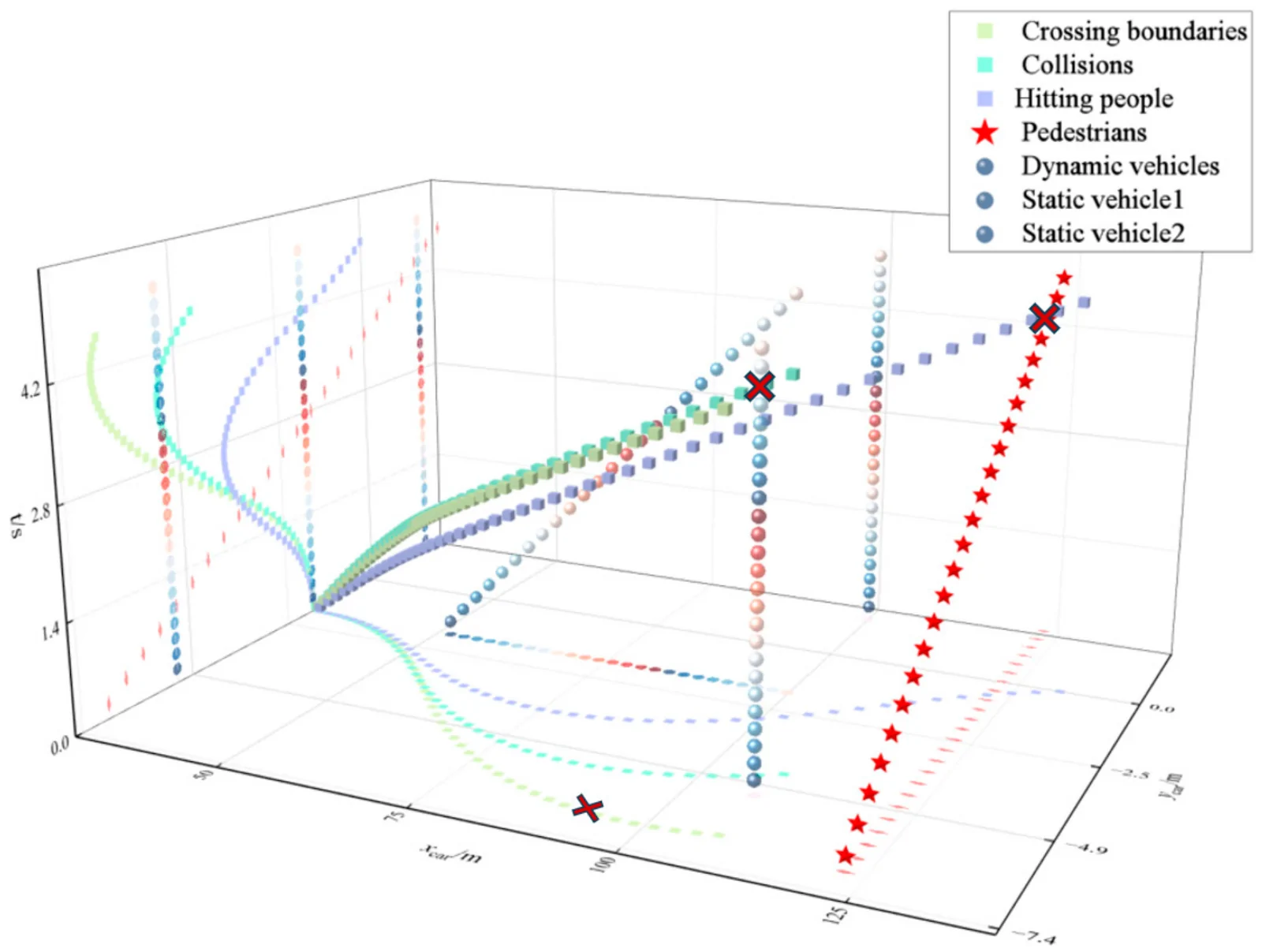
Motion trajectory diagram of DDPG with short-term training. The axes denote time *t* in seconds, vehicle longitudinal position *x*_car_ in meters, and vehicle lateral position *y*_car_ in meters. The red X indicates the position where collision occurs.

The comparative experiment shows that if the driving tendency module is removed from the architecture, the agent exhibits significant policy flaws during the short-term training phase. As shown in Figure 12, the vehicle frequently demonstrates high-risk behaviors such as trajectory boundary violations, collisions with obstacle vehicles, and conflicts with pedestrians. This result validates the critical role of the driving tendency module in guiding exploration direction and constraining the action space. Its absence directly leads to a lack of effective behavioral preference guidance during training, making it difficult to learn safe and compliant driving policies within a limited number of episodes.

**Figure 13:**
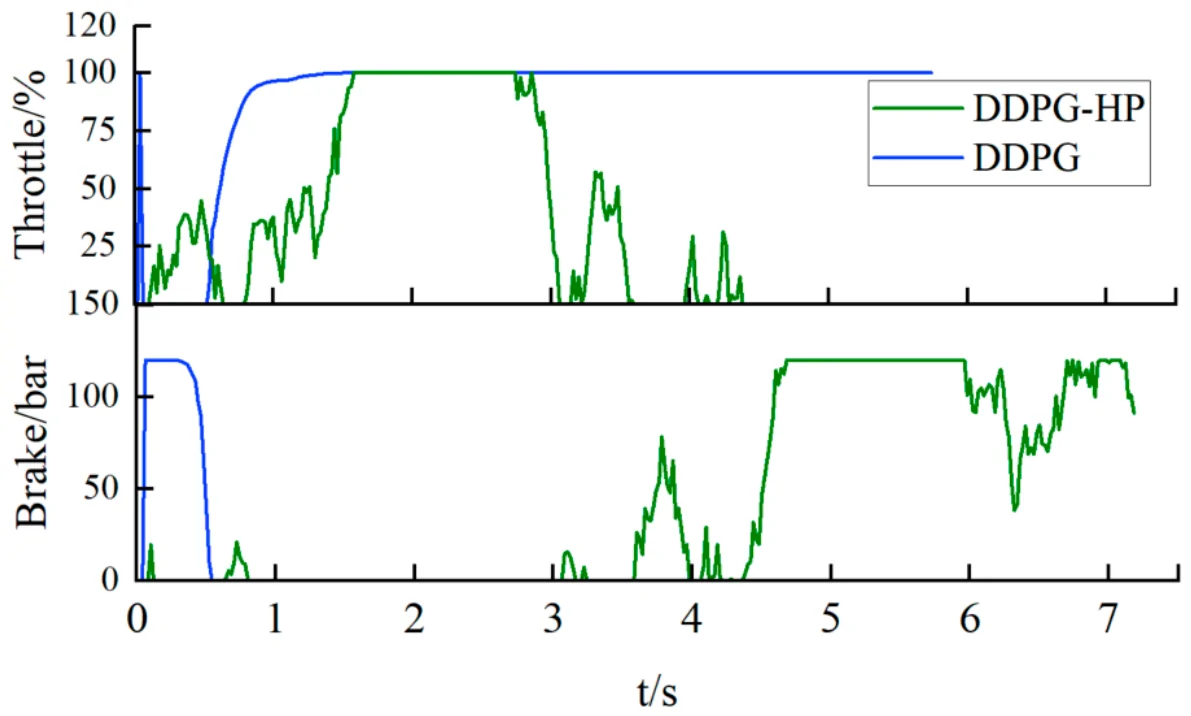
Comparison of Longitudinal Driving Control between DDPG-HP and DDPG.

**Figure 14:**
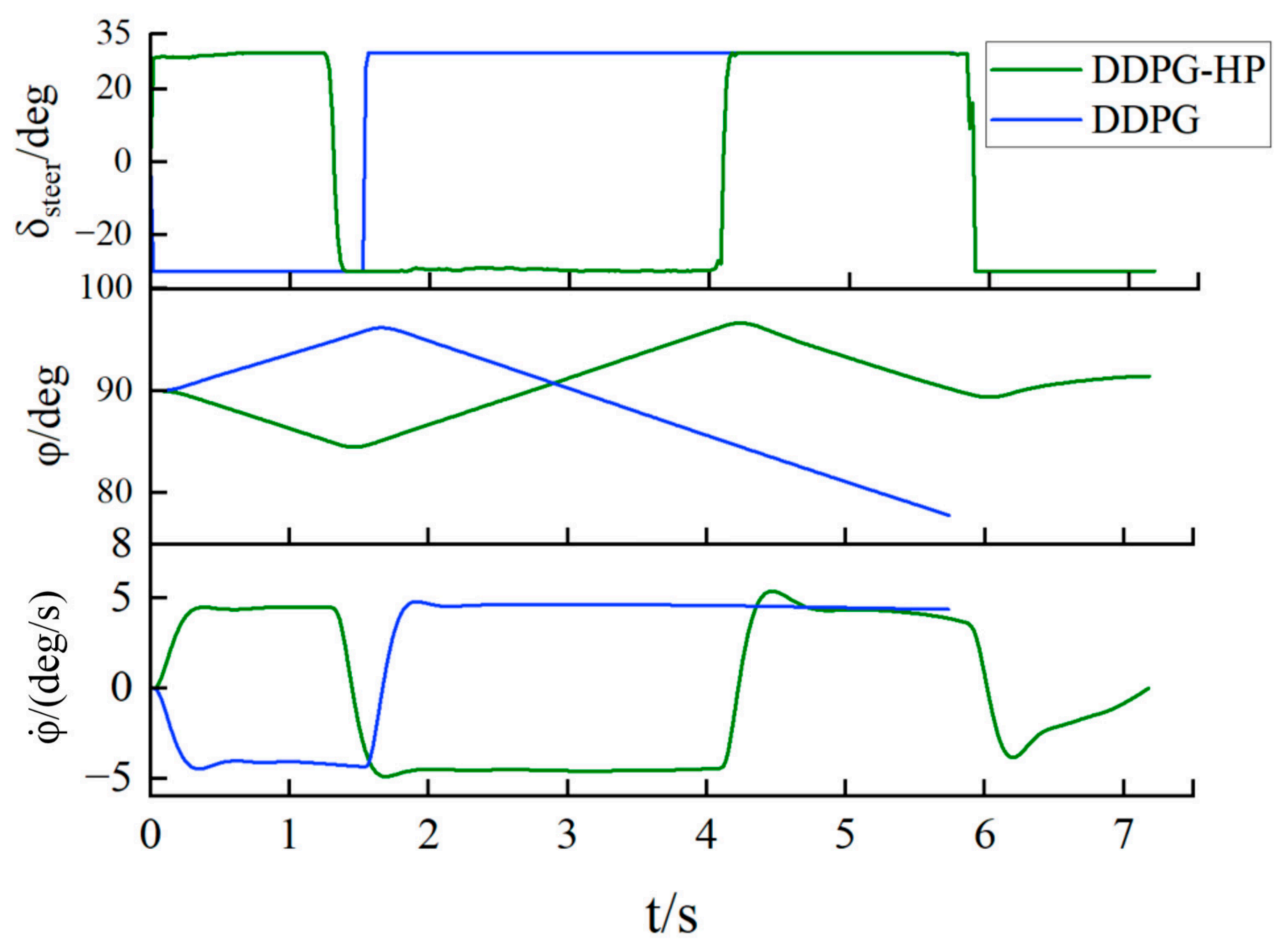
Comparison of Front-Wheel Driving Control, Yaw Angle, and Yaw Rate between DDPG-HP and DDPG.

**Figure 15:**
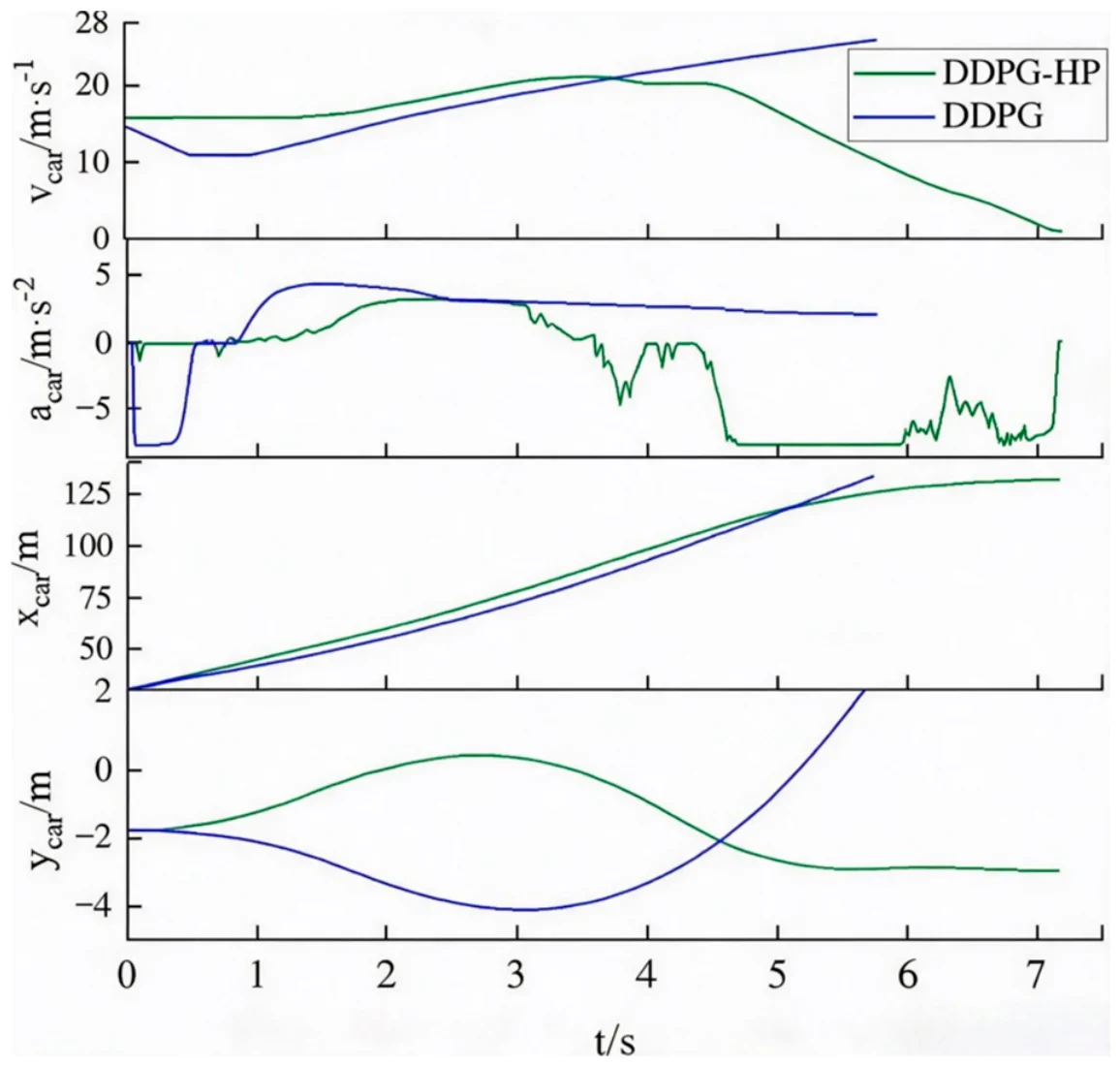
Comparison of Vehicle Motion States between DDPG-HP and DDPG.

Figure 13, Figure 14 and Figure 15 compare the DDPG network trained for 6000 episodes with the DDPG-HP network trained for 3000 episodes.

Compared with standard DDPG, the DDPG-HP algorithm demonstrates advantages in foresight and safety when approaching crosswalks and areas of potential interaction with pedestrians. While effectively avoiding dynamic and static obstacles, it can initiate progressive deceleration in advance. Before nearing obstacle vehicles or pedestrians, the throttle begins to decrease smoothly, and braking is applied gradually through multiple small, continuous interventions. This avoids the abrupt full-throttle or hard-braking phenomena observed in DDPG, resulting in gentler changes in longitudinal acceleration. When encountering potential conflict scenarios, DDPG-HP can complete braking decisions ahead of time, ensuring a safe distance. On normal, unobstructed road sections, it improves overall traffic efficiency through more natural acceleration behaviors, achieving a good balance between safety and efficiency.

The steering angle *δ* output of DDPG-HP is smoother and more continuous, with smaller fluctuations and quicker recovery of the yaw rate during steering maneuvers. When encountering obstacles, the vehicle tends to avoid them while largely maintaining its position near the lane center, and the overall trend of the yaw angle change more closely resembles the continuous adjustments typical of natural human driving. In contrast, DDPG exhibits significant oscillations over short periods. This indicates that the algorithm achieves smoother trajectory during multi-obstacle avoidance.

During driving, the DDPG-HP vehicle maintains more stable lateral offset and more uniform longitudinal position progression, avoiding the sharp acceleration or lateral deviation observed in DDPG in later stages. Particularly in pedestrian crossing and multi-obstacle scenarios, by integrating driving tendency priors, DDPG-HP completes anticipatory steering and speed adjustments in advance, significantly increasing the safety margin before potential conflict points, substantially reducing collision risk.

**Figure 16:**
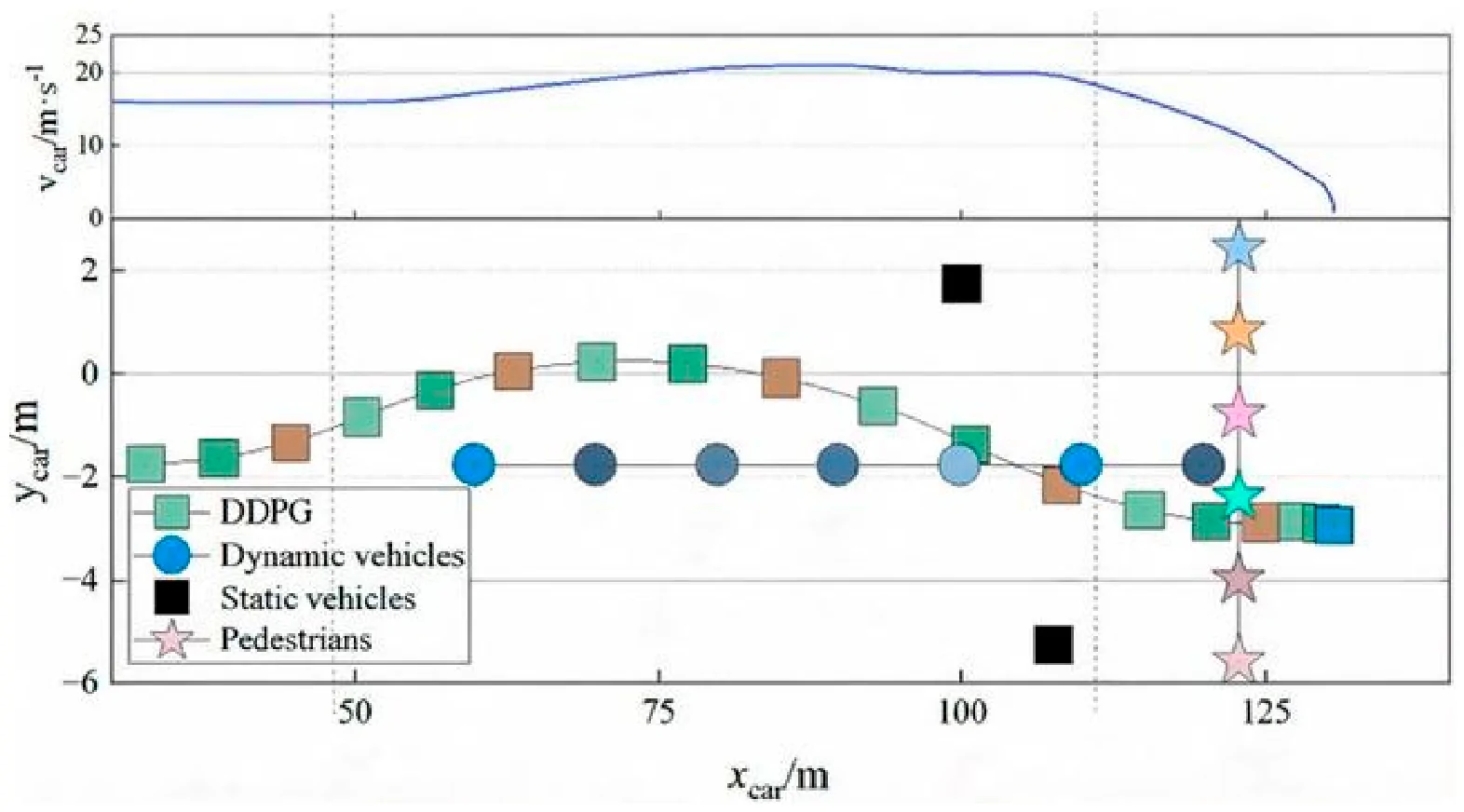
Driving Environment and Trajectory Schematic of DDPG-HP. Different markers and colors in the plot denote the ego-vehicle trajectory, dynamic vehicles, static vehicles and pedestrians, respectively.

In summary, compared with the baseline methods, the DDPG-HP algorithm not only achieves high-priority protection for pedestrian safety while avoiding dynamic and static obstacles but also demonstrates advantages in control smoothness, ride comfort, and decision-making foresight. This shows how human driving experience priors can both constrain and improve reinforcement learning policies. A schematic of the vehicle’s driving environment and trajectory is shown in Figure 16.

To comprehensively evaluate the overall performance of the DDPG-HP algorithm, this study introduces an additional comparative scenario: decoupling lateral path planning and longitudinal speed control to construct a hierarchical control architecture.

In this architecture, the upper layer employs an improved RRT* algorithm for real-time path planning. The inflated collision zone consists of a hard constraint that lies inside obstacles and a soft constraint that lies in a Gaussian decay field, which together ensure the safety of the obstacle avoidance trajectory. This is combined with the Pure Pursuit tracking algorithm to achieve precise control of the front-wheel steering angle. The lower layer uses the DDPG algorithm as the longitudinal controller, optimizing speed tracking strategies through learning to ensure vehicle safety and comfort in pedestrian crossing scenarios.

(1) Hard Constraint

If a sample point lies within the rectangular bounding box of an obstacle for vehicles, $P_{x}\in \left[x_{i}-\frac{L_{obs}}{2},x_{i}+\frac{L_{obs}}{2}\right]$ and $P_{y}\in \left[y_{i}-\frac{W_{obs}}{2},y_{i}+\frac{W_{obs}}{2}\right]$; for pedestrians, $P_{x}\in \left[x_{i}-\frac{L_{\mathrm{ped}}}{2},x_{i}+\frac{L_{\mathrm{ped}}}{2}\right]\;$ and $P_{y}\in \left[y_{i}-\frac{W_{\mathrm{ped}}}{3},y_{i}+\frac{2W_{\mathrm{ped}}}{3}\right]$. Moreover, the cost *C_h_* is set to 128.

where *L*_ped_ = 1 m, *w*_ped_ = 3 m, *L*_obs_ = 4.0 m, *w*_obs_ = 2.1 m.

(2) Soft Constraint

A two-dimensional Gaussian cost field is formed around the obstacle as

(26) $$U_{i}(p_{x},p_{y})=C_{max}\cdot \exp\left[-\left(\frac{{\left(p_{x}-x_{i}\right)}^{2}}{2\sigma_{x,i}^{2}}+\frac{{\left(p_{y}-y_{i}\right)}^{2}}{2\sigma_{y,i}^{2}}\right)\right]$$

**Figure 17:**
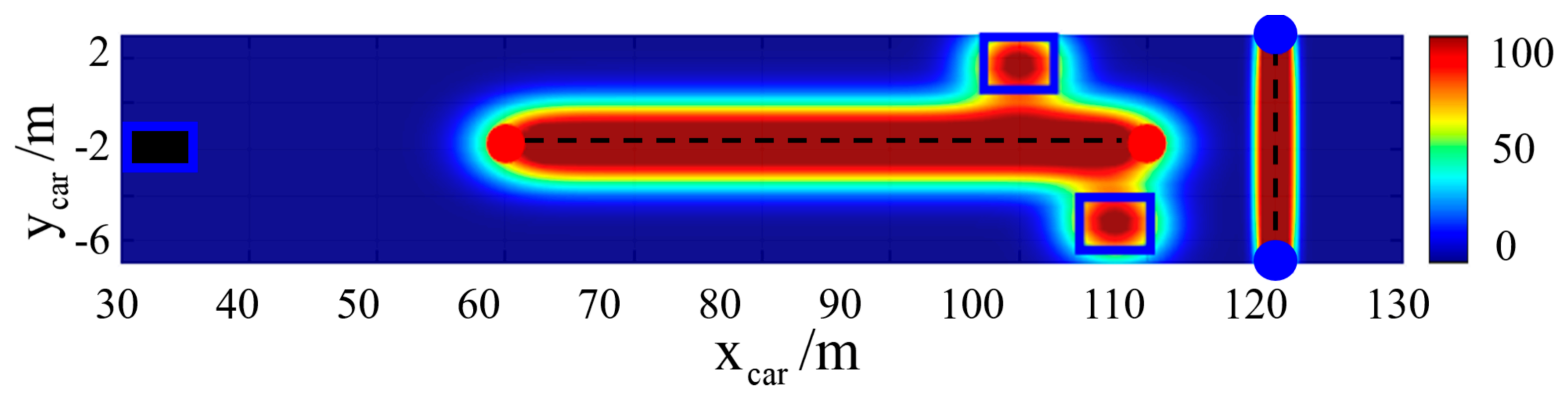
Obstacle Inflation Region Diagram of DDPG-RRT*.

In summary, the total cost field *U* is

(27) $$C(p_{x},p_{y})=\min\left[C_{max},\;\sum_{i=1}^{N}(C_{h,i}(p_{x},p_{y})+U_{i}(p_{x},p_{y}))\right]$$

where (*p_x_*, *p_y_*) are the sample point coordinates, and σ selects the obstacle type which contains vehicle and pedestrian. The inflated zones are shown in Figure 17 below.

**Figure 18:**
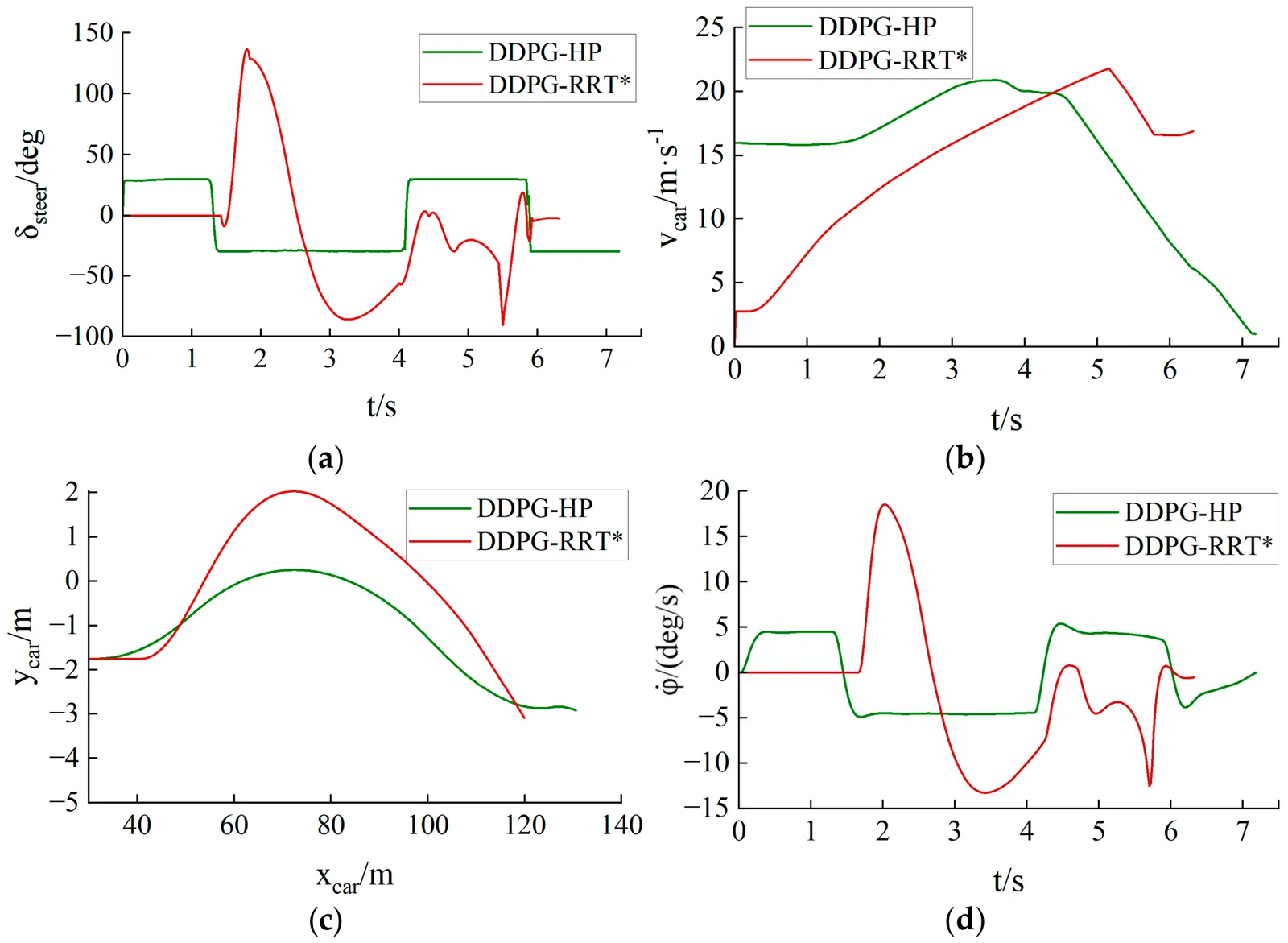
Comparison of Motion Control Performance between DDPG-HP and DDPG-RRT*. (**a**) Comparison of Vehicle Driving States between DDPG-HP and DDPG-RRT*. (**b**) Comparison of Speed Control between DDPG-HP and DDPG-RRT*. (**c**) Comparison of Front-Wheel Drive Control between DDPG-HP and DDPG-RRT*. (**d**) Comparison of Yaw Rate between DDPG-HP and DDPG-RRT*.

**Figure 19:**
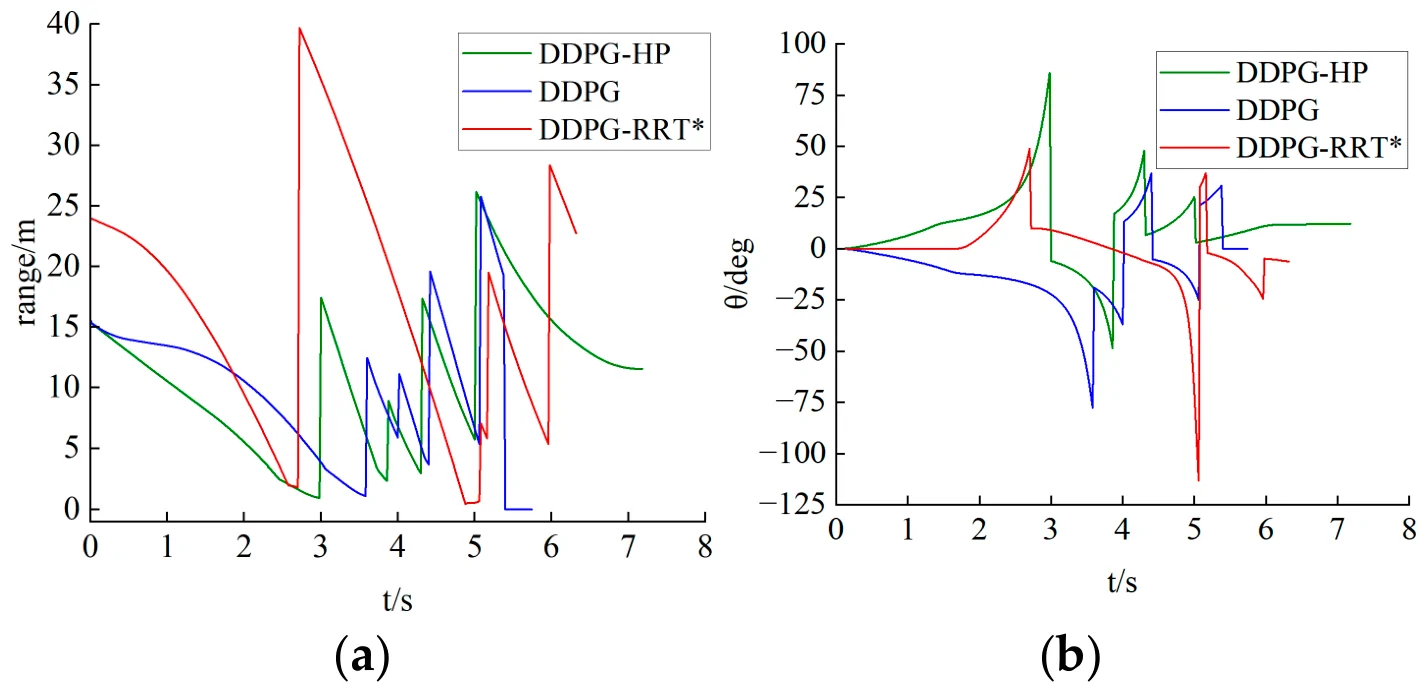
Radar Data Comparison of DDPG-HP, DDPG, and DDPG-RRT*. (**a**) Straight-line distance from obstacle to radar. (**b**) Azimuth angle of obstacle detected by radar.

A comparison of position, yaw angle, yaw rate, and sensor states between the DDPG-RRT* and DDPG-HP methods is shown in Figure 18 and Figure 19.

As shown in Figure 18, compared with DDPG-RRT*, the DDPG-HP architecture demonstrates better comprehensive control performance. During the 7.2 s simulation, it achieved smooth speed regulation: after the initial acceleration phase, it reached peak speed at 3.7 s, then gradually reduced speed to approximately 1 m/s through progressive deceleration, effectively yielding to pedestrians and prioritizing their crossing. This ensures pedestrian safety while enhancing ride comfort. Moreover, DDPG-HP initiates deceleration approximately 1.7 s earlier than DDPG-RRT*, providing a larger safety margin for pedestrian crossing. In terms of lateral control, the steering commands output by DDPG-HP are continuous and smooth, matching the path curvature well. The steering amplitude is effectively constrained within ±30°, and the steering wheel quickly returns to center after completion, exhibiting natural lateral handling characteristics. The yaw rate is also controlled within ±5 deg/s, with stable steering response and no significant overshoot, confirming good lateral stability and trajectory tracking accuracy in high-dynamic scenarios.

In contrast, the DDPG-RRT* architecture exhibits several control limitations in the same scenario. This architecture shows relatively aggressive acceleration behavior from 0 to 5.2 s, reaching a peak of 22 m/s, and experiences significant fluctuations during the subsequent deceleration phase, only reducing speed to 17 m/s. This results in excessively high speed at the crosswalk section, posing risks to pedestrian safety. In terms of lateral control, the steering commands of DDPG-RRT* require frequent adjustments, with steering angle peaks even exceeding ±100° in the intervals of 1–3 s and 5–6 s. This reflects coupling oscillations between path planning and tracking control in its hierarchical structure, leading to noticeable over-correction.

The simulation shows that DDPG-HP can maintain a relatively stable target distance within the 8 s simulation cycle, with a minimum of 1.4 m. At this point, the ego vehicle passes over the obstacle vehicle, retaining a safe distance between the two vehicles. In comparison, DDPG-RRT* experiences a situation where the obstacle vehicle is too close to the radar around 5 s, with a minimum distance of 0.5 m from the obstacle, posing a collision risk. Although the baseline DDPG is relatively stable overall, it comes too close to pedestrians in the later stage.

**Figure 20:**
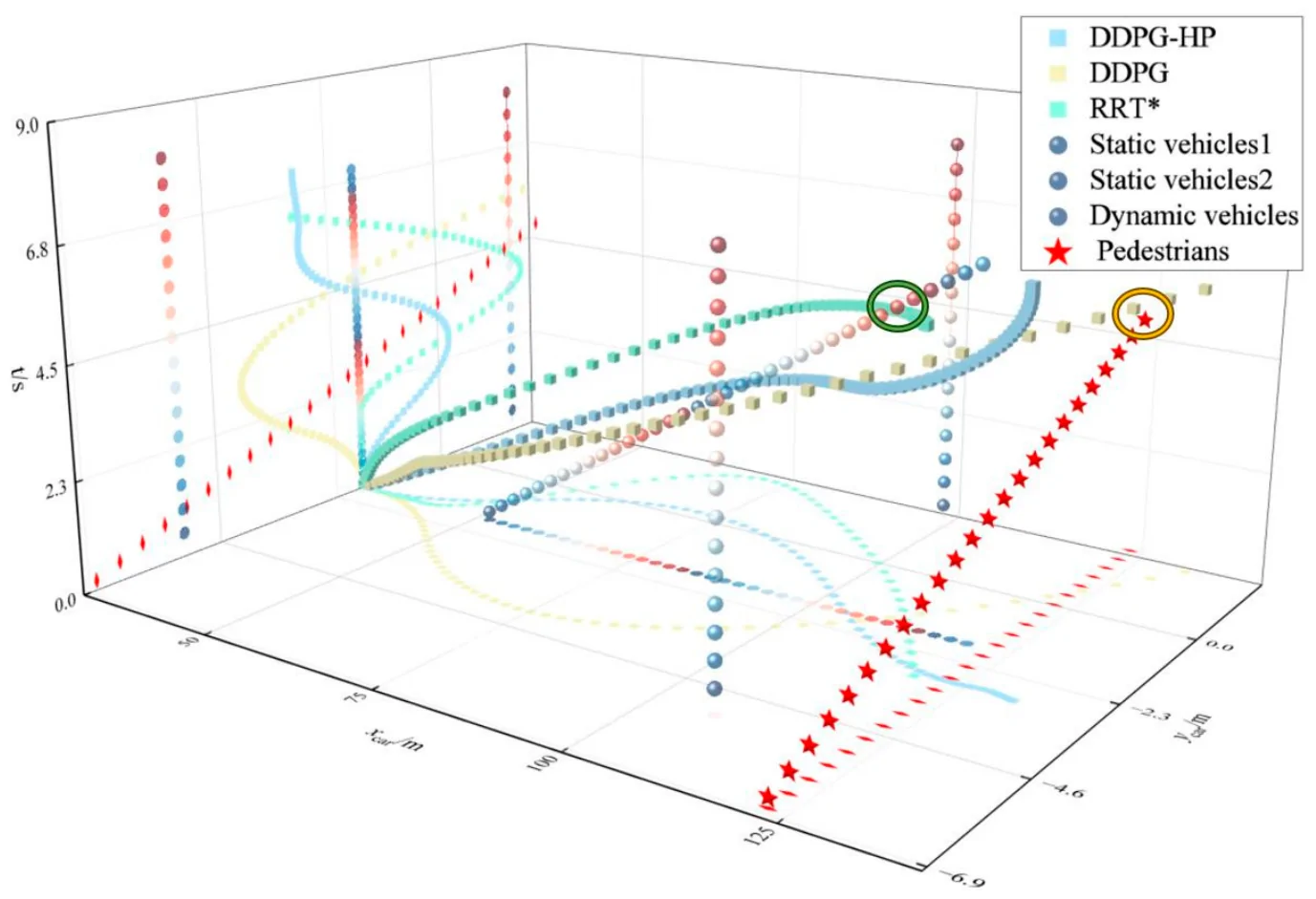
Trajectory Comparison between DDPG-HP, DDPG and RRT* Algorithms. The axes denote time *t* in seconds, vehicle longitudinal position *x*_car_ in meters, and vehicle lateral position *y*_car_ in meters. The green circle and yellow circle in the plot highlight the positions with the highest potential collision risk, which are consistent with the collision locations shown in Figure 12.

In summary, DDPG-HP can maintain a reasonable and stable inter-vehicle distance in following scenarios with small fluctuation ranges. Both DDPG and DDPG-RRT* exhibit sudden distance changes during certain periods, indicating their insufficient response to abrupt environmental changes. In contrast, DDPG-HP achieves more efficient longitudinal speed management through human driving tendency priors, enhancing safety while ensuring traffic efficiency. The trajectory comparison in Figure 20 further illustrates that DDPG-HP maintains a smoother and more stable path than DDPG-RRT* in the multi-obstacle scenario.

## 5. Discussion

Pure reinforcement learning for autonomous driving decision-making faces challenges such as slow convergence, control jitter, and instability in multiple scenarios. To address these issues, this paper proposes a hybrid decision-making framework, DDPG-HP, which integrates V2X communication, human–machine interaction experience, and deep reinforcement learning. Through comparative simulations with baseline algorithms lacking the driving tendency module and the hierarchical planning-based DDPG-RRT* method, the results show that the proposed end-to-end autonomous driving decision-making and control framework achieves performance advantages in complex dynamic traffic scenarios.

The system based on the Actor–Critic dual-network structure performs well in high-dynamic interaction scenarios such as pedestrian crossings. By incorporating prior knowledge of human driving tendencies, this framework can not only cooperatively avoid dynamic and static obstacles but also achieve anticipatory, progressive deceleration before crosswalks, thereby significantly enhancing pedestrian safety. Its lateral control output is smooth and continuous, and although there are minor fluctuations in longitudinal acceleration, it overall effectively reduces collision risks in pedestrian interaction scenarios. Furthermore, to address abnormal conditions in V2X communication, this paper designs a state compensation mechanism based on reverse smoothing Kalman filtering. Utilizing vehicle kinematic priors, this mechanism effectively suppresses state jumps and perceptual noise during communication packet loss or delays. It should be noted that this compensation performance is validated with the simplified V2X anomaly model described in Section 3.6, and its effectiveness under more complex real-world communication conditions requires further investigation. Experiments show that the proposed method enhances the system’s adaptability and robustness under the simplified V2X anomaly model used in this study.

These simulation results confirm the safety and robustness of the DDPG-HP framework in multi-obstacle scenarios. The validation is based on a co-simulation platform that incorporates vehicle dynamics, sensors, and V2X communication. Extending the validation to HIL testing, real-vehicle experiments, and comparisons with a broader range of reinforcement learning baselines such as SAC and PPO would provide further insight into generalization and engineering applicability. Due to experimental constraints, a sensitivity analysis or per-weight ablation of the adaptive weighting mechanism was not performed, which is a limitation of the current study and will be addressed in future work. In summary, through structural design and mechanism innovation, the DDPG-HP framework achieves systematic improvements in decision-making safety, smoothness, and environmental adaptability. Compared with standard DDPG and DDPG-RRT*, it demonstrates advantages in convergence speed and control performance. The framework’s real-time performance and robustness are validated in the co-simulation environment that incorporates a packet-loss and delay model, providing an effective solution for autonomous driving decision-making in high-dynamic, strongly interactive scenarios.

## Data Availability

The original contributions presented in the study are included in the article. Further inquiries can be directed to the corresponding author.

## Abbreviations

The following abbreviations are used in this manuscript:

DDPG	Deep Deterministic Policy Gradient
HP	Human Preference
V2X	Vehicle-to-Everything
RRT*	Rapidly exploring Random Tree star
OU	Ornstein–Uhlenbeck
TTC	Time to Collision
MDP	Markov Decision Process
ReLU	Rectified Linear Unit
FPS	Frames Per Second
HIL	Hardware-in-the-Loop
SAC	Soft Actor–Critic
PPO	Proximal Policy Optimization
